# Weaving DNA strands: structural insight on ATP hydrolysis in RecA-induced homologous recombination

**DOI:** 10.1093/nar/gkz667

**Published:** 2019-08-02

**Authors:** Benjamin Boyer, Claudia Danilowicz, Mara Prentiss, Chantal Prévost

**Affiliations:** 1 CNRS, Université de Paris, UPR 9080, Laboratoire de Biochimie Théorique, 13 rue Pierre et Marie Curie, F-75005 Paris, France; 2 Presently in Laboratoire Génomique Bioinformatique et Applications, EA4627, Conservatoire National des Arts et Métiers, 292 rue Saint Martin, 75003 Paris, France; 3 Department of Physics, Harvard University, Cambridge, MA 02138, USA; 4 Institut de Biologie Physico-Chimique-Fondation Edmond de Rothschild, PSL Research University, Paris, France

## Abstract

Homologous recombination is a fundamental process in all living organisms that allows the faithful repair of DNA double strand breaks, through the exchange of DNA strands between homologous regions of the genome. Results of three decades of investigation and recent fruitful observations have unveiled key elements of the reaction mechanism, which proceeds along nucleofilaments of recombinase proteins of the RecA family. Yet, one essential aspect of homologous recombination has largely been overlooked when deciphering the mechanism: while ATP is hydrolyzed in large quantity during the process, how exactly hydrolysis influences the DNA strand exchange reaction at the structural level remains to be elucidated. In this study, we build on a previous geometrical approach that studied the RecA filament variability without bound DNA to examine the putative implication of ATP hydrolysis on the structure, position, and interactions of up to three DNA strands within the RecA nucleofilament. Simulation results on modeled intermediates in the ATP cycle bring important clues about how local distortions in the DNA strand geometries resulting from ATP hydrolysis can aid sequence recognition by promoting local melting of already formed DNA heteroduplex and transient reverse strand exchange in a weaving type of mechanism.

## INTRODUCTION

Homologous recombination (HR) is a fundamental biological process common to all living organisms. It enables both the faithful repair of DNA double strand breaks, necessary for cell survival, and the crossover of endogenous genes or insertion of exogenous genes, which promotes diversification and species survival in the long term (1–3). These functions are performed in the cell within long helical nucleoprotein filaments formed by the polymerization of recombinase proteins (RecA for prokaryotes) on a single-stranded DNA (ssDNA, also called incoming strand) resulting from the processing of damaged DNA (4). As will be discussed below, ATP is hydrolyzed during the process.

In their active form recombinase filaments can locally incorporate double-stranded DNA (dsDNA) from the genome, check sequence homology with the bound single strand, and promote strand exchange if the degree of homology is sufficient. Kinetics studies have shown that initial stages of DNA recognition and pairing exchange do not depend on ATP hydrolysis (5–7). Indeed, the time scale of ATP hydrolysis in recombination filaments exceeds by more than two orders of magnitude that of these initial HR stages, in the order of milliseconds, which means that hundreds of initial search events can occur in a given region of the filament in the interval between two successive hydrolysis events. We have shown that tension in the dsDNA strands and a series of kinetic decisions play prominent roles to account for the very short time scales of these initial stages (8,9). Intriguingly, while ATP hydrolysis influences the kinetics of subsequent steps (6), hydrolysis is not necessary for the whole HR reaction to successfully go to completion *in vitro* on limited lengths of homologous DNA up to few kilobases (10).

Yet, RecA in its active filament form hydrolyzes large quantities of ATP (11). ATP hydrolysis mostly occurs when DNA is bound to the filament, either as a single-strand, a double-strand, or during strand exchange. The rate of hydrolysis varies with the number of bound DNA strands from 20 min^−1^ (dsDNA or strand exchange) to 30 min^−1^ (ssDNA) (12,13). In physiological conditions, only a fraction of ATP molecules bound to the RecA filament is simultaneously hydrolyzed. Although ATP hydrolysis is known to be involved in the dissociation of terminal RecA monomers, most hydrolysis events occur in monomers distributed all along the filament (11) that generally do not dissociate (14).

ATP hydrolysis has been found responsible for a number of characteristics of the HR process such as the filament disassembly after strand exchange completion or the possibility of reverse exchange reaction (4,15–17). More recently, evidence was found that ATP hydrolysis directly influences the dynamics of strand exchange: when ATP is hydrolyzed, strand exchange propagation proceeds within sliding DNA windows with lengths of ∼80 bp (18,19), whereas the turnover rate of the dsDNA that is searched for sequence match increases (20). In addition, our recent results indicate that in the presence of ATP hydrolysis, the formation of heteroduplex segments with length comprised between 20 and 75 bp remains highly reversible, insensitive to the segment length (19). Even fully homologous segments can rapidly interrupt strand exchange and return to reactants. In contrast, in the presence of ATPγS, a non hydrolyzable ATP homolog, the stability of the newly formed strand exchange product strongly increases with length: segments with lengths >40 bp are almost never observed to unbind.

Combined together, these observations provide little indication of what exactly happens at the structural level that would transform the chemical energy released by ATP hydrolysis into a mechanical action accounting for the specificities of the ATP driven HR process. The major structural information concerns the nucleotide-dependent preferred geometries of the HR filament, extended in the presence of ATP or non-hydrolyzable analogs (active filament, pitch value ≈ 85–100 Å) (21) and compressed in the presence of ADP or with no bound cofactor (pitch value ≈ 65–85 Å) (22). The two forms differ by the orientation of the monomers with respect to the axis and by distinct monomer-monomer binding geometries (21,23,24). Notably, the ATP cofactor is buried in the interface between adjacent monomers in the extended form, while it is accessible to the solvent in the compressed form. At the local level, quantum chemical studies indicate a reorganization of the hydrogen-bond network at the protein–DNA interface (25).

Three possible structural responses to hydrolysis have been envisioned. In the so-called redistribution hypothesis (26,27), hydrolysis of ATP bound to a given monomer prompts that monomer to unbind, exchange its ADP cofactor for a new ATP cofactor, and rebind at the filament extremity. This hypothesis did not find experimental support, as only terminal monomers were observed to unbind (14,28,29). In a second scenario, monomers with hydrolyzed ATP conserve the binding geometry they have in the active filament but this geometry is frustrated. Klapstein and Bruinsma proposed an elegant physical model where this frustration would transfer into a rotational movement of the DNA strands (30). There is, however, no structural clue about a mechanism for such chemical to rotational energy transfer. The third hypothesis postulates that monomers switch their binding geometries in the filament from ATP- to ADP-favorable interfaces in response to ATP hydrolysis (24,31). They can then exchange the bound ADP for a new ATP and perform reverse interface switching, thus completing the ATP cycle. In spite of the large monomer displacement (∼20 Å) that accompanies the passage from ATP- to ADP-binding geometries (24), this hypothesis is supported by several *in vitro* experimental observations. First, in the absence of a regeneration system, consumption of all ATP molecules results in the recombination filament cooperatively adopting the compressed form, without any monomer dissociation (29,32,33). When provided with additional ATP or under variable stretching load, filaments were observed to cooperatively cycle between extended and compressed forms. This implies that each monomer modifies its binding geometry and indeed, filaments with mixed binding geometries could be observed in particular conditions (23,34). Individual switch of monomer binding geometries as a response to ATP hydrolysis is also consistent with hydrolysis-related appearance of disorder, observed in ATP regeneration conditions in the otherwise very stiff filaments (35,36). Besides, other systems have been documented where monomers individually modify their binding interfaces. In cyclic systems such as the DnaB (37) or the T7 gp4 helicases (38), coexistence of different interfaces within a same ring-shaped oligomer was associated to sequential stages in the hydrolysis process (38,39). More specifically, multimeric RecA-like domains generally move relative to each other during the hydrolysis cycle (40).

The question of how hydrolysis propagates along the filament was raised. The previously mentioned cooperative cycling between extended and compressed filaments evoke a possible mediation of hydrolysis progression *via* interactions between adjacent monomers (29). Cooperation between adjacent monomer is also implicit in the hydrolysis wave model proposed by the Cox group; however, this model also requires cooperation between non-adjacent monomers (4). Progression of hydrolysis waves along the filament with a spatial periodicity of one helix turn has been detected when more than one DNA strand was present in the HR filament (41). When only one strand was present, the observed distribution of hydrolysis events was random. It has been proposed that different regimes of hydrolysis propagation may develop depending on the level of available free ATP in the environment (29). It is also possible that different propagation regimes occur in different stages of the HR process.

We previously investigated filament architectures in RecA systems that include mixtures of interfaces with ATP binding and interfaces with ADP binding, thus mimicking different distributions of ATP hydrolysis events (24). The present work builds upon that preliminary study to investigate the compatibility of an irregular filament geometry, featuring one ADP-binding geometry in the center of two turns of extended filament, with the binding of up to three DNA strands. The strands successively fill filament sites I (incoming strand, heteroduplex) and II (outgoing strand), therefore exploring the possibility that such structure may exist in the presynaptic, synaptic, and post-synaptic states of the HR process. The stability of these models of irregular DNA-bound filaments is challenged with all-atom MD simulations in solvated environment, revealing the possibility of local reverse pairing exchange that may explain why destabilization of the strand exchange product commonly occurs in the presence of ATP hydrolysis (15,19,42). In addition, examining the stability of ADP-like interfaces in active filaments provides information about possible cooperative assistance to binding geometry changes between neighboring or distant monomers.

## MATERIALS AND METHODS

### Model construction with PTools/Heligeom

Construction of two turns of irregular RecA oligomers with bound DNA was performed using the PTools/Heligeom library and associated scripts (24,43,44). This program relates monomer-monomer binding geometries to the geometry of large oligomeric assemblies. In this work, two specific RecA-RecA binding geometries were used. The first one, issued from the crystal structure of extended RecA filaments in the presence of DNA and non-hydrolyzable ATP analog (PDB entry 3CMW) (21), will be referred to as ATP-binding geometry. The second one, extracted from the crystal structure of a compressed RecA filament in the presence of ADP (PDB entry 2REB) (22), will be referred to as ADP-binding geometry. We note that the terms ATP/ADP-binding indicate a binding mode preference in the presence of ATP/ADP and do not imply that this geometry may exclusively be associated with ATP/ADP binding. For example, the ADP-binding geometry has also been observed in the absence of any cofactor (22).

A RecA filament made of twice the succession of five ATP-bound monomers and one ADP-bound monomer was constructed using the following protocol. First, a regular hexamer made of six RecA monomers (numbered from (*i* − 5) to (*i*) in the direction that corresponds to the 5′-3′ orientation of a single-stranded DNA bound in site I) was built from two consecutive central monomers of the extended crystal structure (3CMW) using the heligeom.py script of the PTools library. We will refer to this structure as a regular filament. The hexamer was then submitted to the screw transformation associated to the ADP-binding geometry, with the transformed hexamer being numbered from (*i* + 1) to (*i* + 6). The transformation resulted in the formation of a 20.5° kink between the axial direction of monomers (*i* − 5) to (*i*) and that of monomers (*i* + 1) to (*i* + 6). The N-terminal region of monomer (*i* + 1) (residues [1–37]) was substituted by the corresponding region taken from the compressed filament (2REB) after superposition of the structurally close protein regions (24). The two flexible loops L1 and L2 of monomer (*i* + 1) (residues [157–164] and [194–210], respectively), which are disordered in the compressed filament structure, were attributed the geometry they present in the extended filament. This produced a dodecameric filament with all interfaces in the ATP-binding geometry, with the exception of the interface between monomers (*i*) and (*i* + 1). This interface presents an ADP-binding geometry.

Three DNA strands were then successively added, with sequence (dT)_36_ for the initiating and outgoing strands and (dA)_36_ for the complementary strand. To this aim, trinucleotide segments respectively taken from the crystal structures 3CMW and 3CMX for the single strand and heteroduplex in site I and from our recent model (8) for the outgoing strand in site II were successively added to the filament in such a way as to conserve their reference binding geometry with each RecA monomer. Disruption of the DNA backbone continuity resulting from the perturbation of the filament geometry near monomer (*i*) was corrected via energy minimization using the NAMD 2.10 software (45,46).

### Molecular dynamics simulation

Molecular dynamics (MD) simulations were performed on each dodecameric filament bound to single-stranded, double-stranded or three-stranded DNA using the NAMD 2.10 software (45) and the CHARMM 27 force field including the CMAP correction (46). The structures were first solvated using a TIP3P water model in a 140 × 144 × 225 Å^3^ box, submitted to periodic boundary conditions. Electric neutrality and a physiological ionic concentration of 0.15 mol/l were obtained by the addition of respectively 438, 469, 503 Na^+^ and 353, 349, 348 Cl^−^ ions for the single-stranded, double-stranded, and three-stranded systems. The corresponding electric charge before neutralization was –85, –120, –155 e^−^. As a whole, the simulated systems contained between 435 000 and 439 000 atoms. After 5000 steps of conjugate gradient energy minimization, the system was progressively heated to 300 K. A 30-ns equilibration phase was followed by 100-ns production. The three-stranded system was submitted to three independent simulations using identical protocols. In addition, a 100-ns simulation was independently performed starting from a three-stranded system with two artificially reversed base pairings. Time steps were set to 2 fs using the SHAKE algorithm, and long-range electrostatic interactions were accounted for using the particle-mesh Ewald method. Van der Waals interactions were smoothly switched off between 10 and 12 Å. The temperature and pressure were maintained using a Langevin dynamics scheme and a NosHooverLangevin piston, respectively. During equilibration, the positions of the protein C_α_ carbons were harmonically restrained to their initial position, with force constant decreasing from 0.5 to 0.05 kcal.mol^−1^.Å^−2^. No restraints were applied during the production phase. Analysis used in-house python scripts based on the PTools library (24,43) together with VMD tools (47). Notably, the width of the groove entrance at the level of a given monomer (*k*) was calculated as the smallest distance between the C-terminal domain of that monomer and the N-terminal domains of the three closest monomers across the groove (monomers (*k* + 5), (*k* + 6) and (*k* + 7)). This definition differs from the rigorous groove width definition described in Boyer *et al.* (24) that only applies to regular helical filaments.

## RESULTS

In the presynaptic RecA filament built on ssDNA as well as in filaments undergoing strand exchange, ATP is constantly hydrolyzed (41). In order to explore the structural implication of isolated hydrolysis events occurring within RecA filaments, we considered filaments where at a given moment one unique hydrolysis event occurs within a helical turn and two consecutive hydrolysis spots are separated along the filament by at least five monomers with bound ATP. Possible consequences of having shorter or longer distances between hydrolysis spots will be addressed in the discussion.

### Stability of the DNA strands in regular RecA filaments

**Figure 1.**
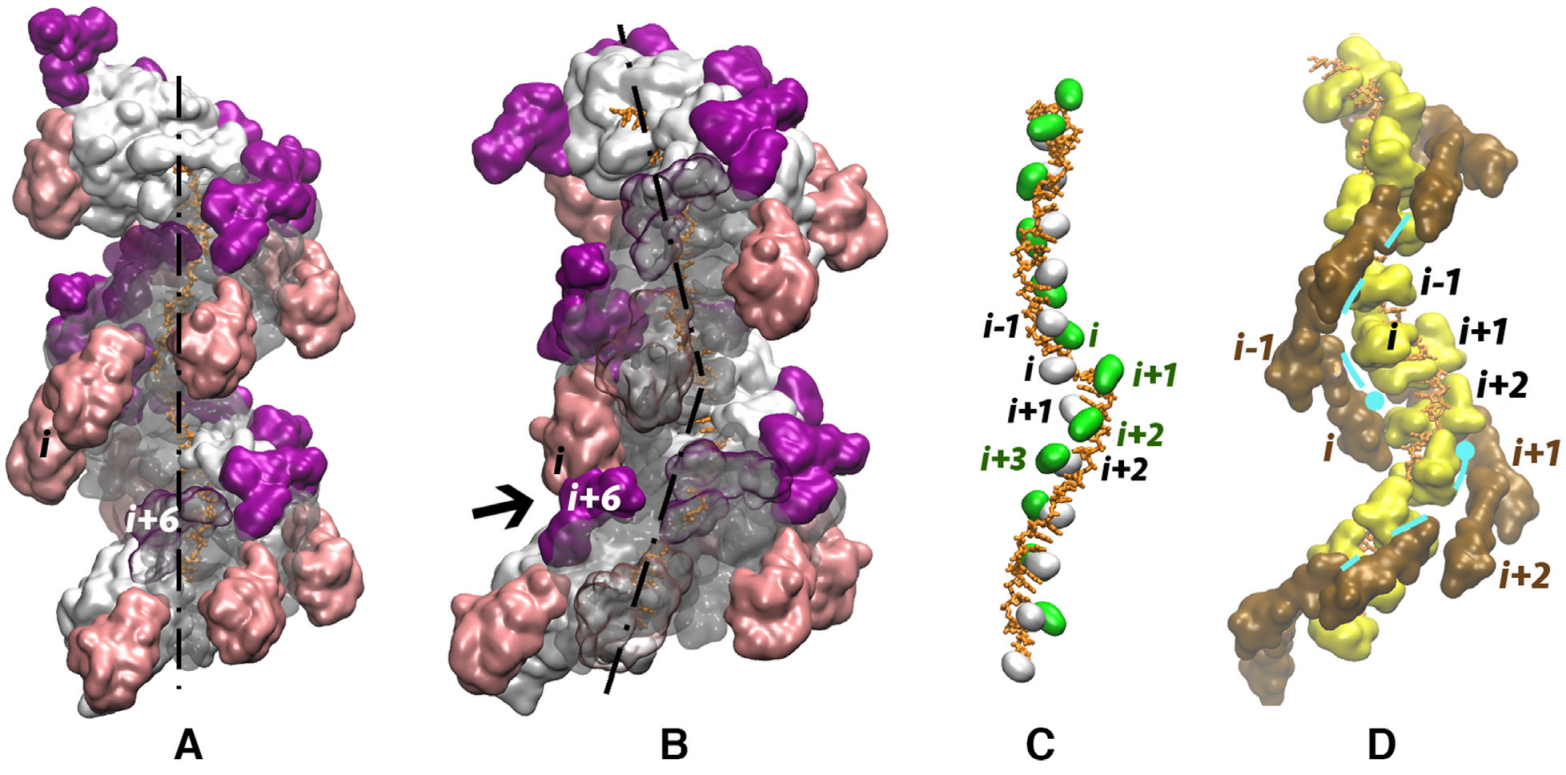
(A, B) Surface view of two turns of RecA filaments built on a DNA single-strand with (**A**) a regular ATP-binding geometry and (**B**) a distorted geometry obtained when replacing the central interface by an ADP-binding geometry. The filaments are represented in white, with the C-terminal domains in pink and the N-terminal domains in magenta. The DNA strand is in orange. Regions of the protein that cover the DNA strand are represented in transparency. In (B), the black arrow points at the close contact between the C-terminal domain of monomer (*i*) and the N-terminal domain of monomer (*i* + 6); a 3D view is available in supplementary information SI-II. (C, D) Details of site I (**C**) and site II (**D**) disorganization in the distorted filament : in (C), Met164 (green, from L1 loop, labelled in green with the corresponding monomer index) and Ile199 (silver, from L2 loop, labelled in black) that form hydrophobic contacts in intercalations sites every three nucleotides are in surface representation; see Supplementary Figure SI-1 for more detail; (D) the binding site II track, that runs between L2 loops (yellow, surface representation, labelled in black with the corresponding monomer index) and LexA-binding loops (marron, surface representation, labelled in marron) is represented as a cyan broken line, and its disrupted extremities are featured with cyan circles. All graphical representations have been made using the VMD software (47).

**Figure 2.**
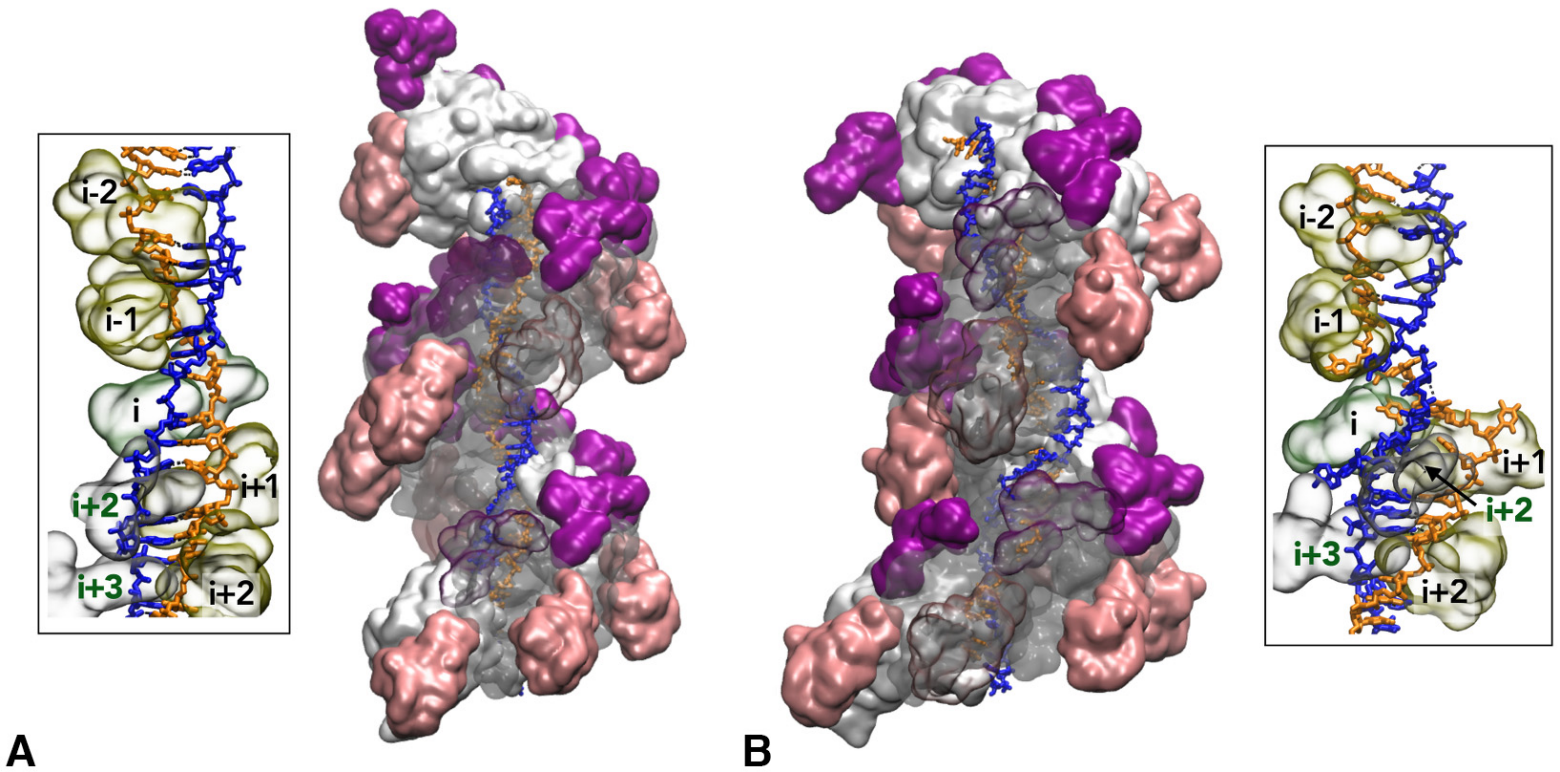
Same as Figure 1A, B but a second DNA strand (complementary strand, in blue) has been added in site I. Inserts represent details of the steric interactions between DNA strand bases and selected protein loops L2 (194–212, labelled with the monomer index, in black) and/or L1 (156–165, monomer index in green) that stabilize the DNA elongation in (**A**) and the distortions in (**B**); loops L1 and L2 are represented as transparent surfaces.

**Figure 3.**
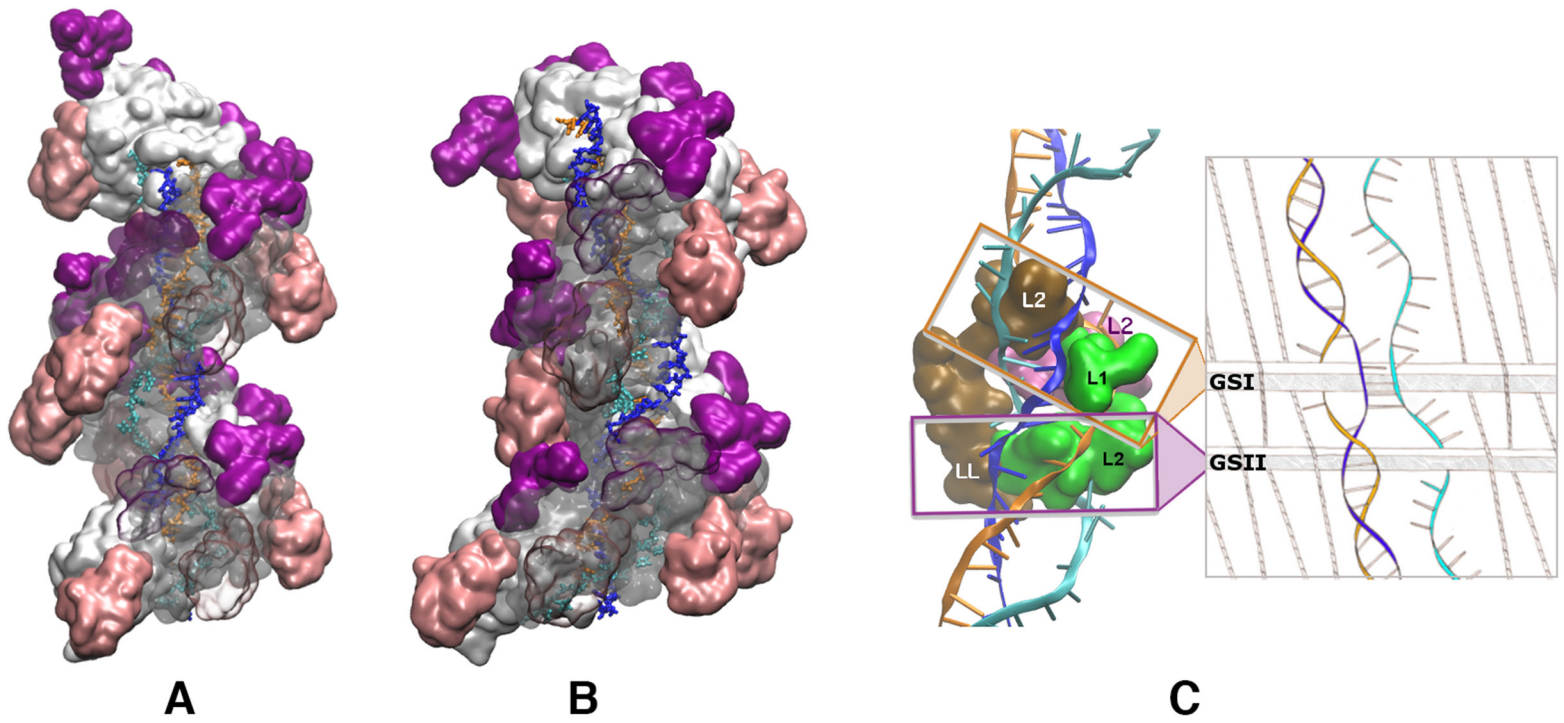
(**A**, **B**) Same as Figures 1 and 2A, B with a third strand (outgoing strand, in cyan) added in site II. (**C**) Analogy between the partitioning resulting from the internal organization of the filament interior (left) and a weaving machine (right scheme); regions of the protein that participate in the strand separation or pairing (GSI and GSII) are represented in surface mode and colored according to the monomer they belong to (monomer (*i*) in marron, (*i* + 1) in pink and (*i* + 2) in green). In 5′, the GSI group formed by the L2 loops of subunits (*i*) and (*i* + 1) and loop L1 of (*i* + 2) separate the two strands of the heteroduplex while in 3′ the tight connection between the L2 loop of (*i* + 2) and LexA-binding loop (labelled LL) of monomer (*i*) (group GSII) ensures the separation between the outgoing strand (cyan) and the complementary strand (blue). Loops L1 and L2 of monomer (*i* + 2) also restrain the complementary strand position in such a way that it stays close to the outgoing strand pathway.

In the extended form of regular RecA filaments, the DNA strands interact with the protein via a mixture of electrostatic and hydrophobic interactions in binding sites I and II (Figures 1–3A) (9,21). Both sites consist of narrow tracks separated by an array of bulky L2 loops (one loop per RecA monomer) and lined with the inside filament wall on the other side (Figure 1C, D). During HR, binding sites I and II, respectively, accommodate the incoming and the outgoing strands, which are stretched by 50% and unwound by 40% with respect to physiological B-form dsDNA. The lateral displacement of binding site II from the filament axis further increases the extension of the outgoing strand backbone, which reaches its upper stretching limit (48). Strong interactions every three phosphate groups with clusters of basic residues Arg226, Arg227, Arg243 and Lys245 stabilize this extremely extended conformation (Figure 3A and supplementary information Supplementary Figure SI-1B) (9). Both sites I and II can accommodate a second strand, although the highly stressed dsDNA in site II cannot extend over more than ten or twelve base pairs without unbinding, unless the complementary strand switches from site II pairing to site I pairing (8). In the present study, we concentrate on the case where the duplex DNA resulting from strand exchange is in site I during the elongation stage where ATP hydrolysis influences the course of the reaction. In regular filaments, this structure is stabilized by the insertion of hydrophobic residues Ile199 (L2 loop) and Met164 (L1 Loop) within large intercalation sites that result from the stretching/unwinding distortions of the DNA (21) (Figure 1C).

### Models of mixed ATP/ADP filaments

Dodecameric filaments (approximately two helical turns) presenting a central ADP-bound monomer (monomer (*i*)) and with up to three bound DNA strands were modeled as described in Methods. The first consequence of the change in monomer (*i*) binding geometry was to extract its ADP cofactor from the (*i*), (*i* + 1) interface and make it accessible to the solvent (22), which may facilitate its unbinding and replacement by ATP. In the mixed filament, the ADP cofactor is however situated in a very crowded environment characterizing the filament interior, described below.

Independently of the number of bound DNA strands, the protein component in all three models showed strong particularities with respect to the regular form of the extended HR filament (Figures 1–3). First of all, introduction of an ADP-like interface within an active filament geometry resulted in modifying the helical axis orientation by 20.5° (24). This brought regions of the filament that are otherwise distant from each other into close proximity. One of the most striking contacts took place between the C-terminal domain of monomer (*i*) and the N-terminal domain of monomer (*i* + 6) (arrow in Figure 1B). This contact, which involves residues 287 to 290, 292, 295 to 297 from monomer (*i*), and 19 to 21 from (*i* + 6), completely closed the filament groove between monomers (*i*) and (*i* + 6) (see 3-D view in supplementary information SII). It stably maintained the groove closed all along the 100 ns MD runs with one, two, or three DNA strands (Supplementary Figure SI-2). The (*i*), (*i* + 6) groove closure was accompanied by a narrowing of the groove entrance at the level of monomers (*i* − 5), (*i* − 4), and (*i* − 1) and some widening with large amplitude variation at the level of monomers (*i* − 3) and (*i* − 2) with respect to the 28 Å value observed in regular crystal structure filaments (as measured by the closest distance between C-terminal domains and N-terminal domains across the groove, see Methods). Other structural elements substantially modified their position inside the filament and occupied regions in space that are vacant in the regular filament: Figure 1D and Supplementary Figure SI-3 show how the bulky LexA-binding loop of monomer (*i*) (residues 225 to 245), the L2 loops of monomers (*i*), (*i* + 1) and (*i* + 2), and the L1 loop of (*i* + 2) contributed to modifying the internal partitioning of the filament groove, *i.e*. the size and spatial organization of cavities the DNA strands can occupy inside the filament. Notably, the narrow tracks that form sites I and II were both locally disrupted. In addition, hydrophobic contacts between L2 loop residue Ile199 of monomer (*i*) and L1 loop-Met164 of (*i* + 1) (see previous section) were lost. We examine below the consequences of these distortions on the structure, interactions, and stability of the incoming strand in site I, the heteroduplex in site I, and the outgoing strand in site II.

### Incoming strand in site I

As stated above, the change in the filament axis orientation between monomers (*i*) and (*i* + 1) in the mixed filament resulted in locally disrupting the interaction network of site I. As a result, the incoming strand backbone formed an abrupt kink that extended over six nucleotides (Figure 1B, C). Specifically, residues Arg169 and Arg176 of monomer (*i* + 1) moved away from site I and could not maintain their regular interactions with the phosphodiester groups (Supplementary Figure SI-1A). New interactions partly compensated for this loss within the distorted region, such as salt bridges involving Arg196 and Lys198 of monomer (*i*) and interactions at the extremities of the kinked region were reinforced. However, the overall distortion increased the L1 loop fluctuations in monomers (*i*) and (*i* + 1) (Supplementary Figures SI-4A, B and SI-5B). L1 loop fluctuations are important since these loops participate in the interface between consecutive monomers (24). In spite of these fluctuations and the loss of interaction between monomer (*i*) and the DNA, no destabilization of the (*i*), (*i* + 1) interface was observed (see Supplementary Figure SI-6).

Although the degree of backbone stretching in the kinked region, as measured by the distance between the terminal phosphates, was comparable to that in the rest of the structure (33–36 Å for six base intervals), the bases in the kinked region were not partitioned in groups of three bases like in the regular structure. Instead, the bases were separated into one duplet and one quadruplet, where bases stacked either as duplets or as a triplet and a single base. This different partitioning is due to a displacement of residue Ile199 of monomer (*i*) with respect to the ssDNA, forbidding its intercalation between base triplets as observed in the rest of the structure. In addition, intercalation of residues Met164 of (*i* + 2), and transiently (*i* + 1), took place every two bases providing new stacking interactions. We note that residues Met164 do not directly intercalate between incoming strand bases in regular structures made on ssDNA (21). As a whole, the combined L1 and L2 loops of monomers (*i*) to (*i* + 2) exerted a close steric control on the position and geometry of the incoming strand kinked region. This is probably the reason why the structure of the incoming strand remained stable over 100 ns MD simulations (see Supplementary Figure SI-7, ‘DNA center’ panel) and after adding the complementary and outgoing strands to the structure that originally contained only the incoming strand (Supplementary Figure SI-8). Noticeably, such steric control, together with the presence of strong hooking interactions with the phosphate groups at both extremities of the distorted region, permitted strand distortions to be confined within the kinked region: outside that region, the incoming strand conserved an intact interaction network with site I.

### Heteroduplex in site I

A strand with sequence complementary to that of the incoming strand was added to obtain a heteroduplex in site I. Formation of the heteroduplex was accompanied by the creation of a six-bp long melting bubble in the kinked region close to monomers (*i*) and (*i* + 1). During the course of the 100-ns MD simulation, the melted region in the complementary strand gradually got closer to the incoming strand (Supplementary Figure SI-9A), however without restoring the lost Watson–Crick interactions (Figure 2B). A new interaction network developed, where bases either formed inter-strand stacking interactions or flipped out of the melted region to insert into protein hydrophobic pockets (Figure 2B, insert). During the reorganization, the complementary strand backbone was sterically confined by the L2 loops of monomers (*i* − 1) and (*i*) on one side and by the L1 loop of monomer (*i* + 2) on the other side. Again, this did not have any impact on the Watson–Crick pairing in the remaining regions of the double helix, which remarkably conserved the interactions in site I that are observed in regular filaments. As will be seen below, adaptation of the complementary strand to the filament distortions showed a strong dependence to the presence of the outgoing strand in site II.

### Heteroduplex in site I and outgoing strand in site II

Similarly to what was observed in site I, the filament distortion at the junction between monomers (*i*) and (*i* + 1) interrupted the helical cavity track between the array of L2 loops and the protein core that corresponds to the secondary DNA binding site (Figure 1 D). In spite of the high extension of its backbone in site II, and in spite of completely (for monomer (*i* + 1)) or partially (for (*i* − 1) and (*i* + 2)) losing anchoring interactions with the basic residues Arg226, Arg227, Arg243 and Lys245, the outgoing strand could fill the discontinuity between site II tracks situated on each side of the (*i*), (*i* + 1) junction, following an extremely constrained pathway across the filament groove (Supplementary Figure SI-1B). The perturbation of the outgoing strand spanned nine bases, which is 50% larger than the six-base perturbation observed in site I. The perturbation is larger because site II is laterally displaced with respect to the filament axis, resulting in an increase in the distance between separated extremities of site II.

**Figure 4.**
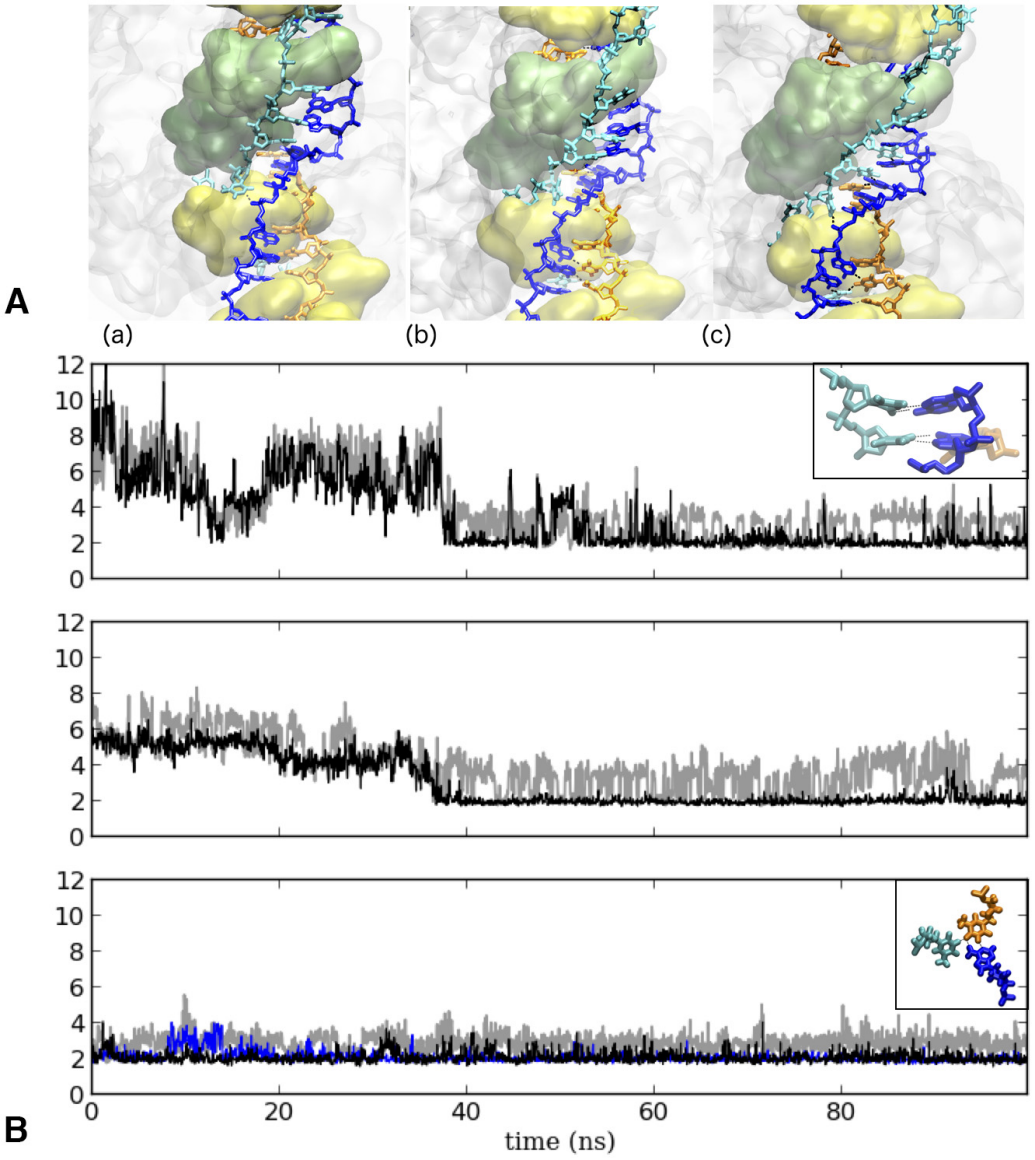
(**A**) Snapshots of the formation of two successive pairing interactions between bases from the complementary strand (blue) and bases from the outgoing strand (cyan) during 100-ns molecular dynamics simulation of the system with three DNA strands; snapshot (a) represents the starting structure, snapshot (b) an intermediate state that occurs during equilibration, and snapshot (c) is the final state. (**B**) Time evolution of pairing distances within these two successive base pairs (top and middle panels) and within one base triplet (bottom panel), all of which spontaneously formed during 100 ns of one of the MD runs; (top and middle) Hoogsteen-type pairing between T17:A16, and T16:A15 where thymines belong to the outgoing strand and adenines to the complementary strand : A:N7–T:H3 (black) and A:H61/2–T:O2 (grey); (bottom) R-type triplet pairing involving one base from each of the three strands : the black line reports the evolution of the shortest distance between (*N1*)T26:O4–(*N2*)A12:H61 and (*N1*)T26:H3-(*N2*)A12:N1, the gray line refers to the (*N2*)A12:H62–(*N3*)T23:O4 distance and the blue line to the (*N1*)T26:O4–(*N3*)T23:H3 distance, where *N1, N2, N3* stand for the incoming, complementary and outgoing strands, respectively; inserts represent the final structure of the paired bases.

In contrast to what was observed in the simulation of the heteroduplex (Supplementary Figure SI-9A), the complementary strand in the three-stranded system mostly remained far away from the incoming strand during four independent MD trajectories (Supplementary Figures SI-9(B-E) and SI-10(g), panels labelled ‘1-2’). Indeed, several bulky regions of the filament including the L2 loops of monomers (*i*) to (*i* + 3), the L1 loop of (*i* + 2), and the LexA-binding loop of (*i*), had their positions restrained by the outgoing strand tension (see e.g. Supplementary Supplementary Figures SI-3B(a), SI-10(b), SI-11). The interplay between these bulky regions not only physically separated the complementary strand from the incoming strand (GSI group in Figure 3C), but also confined the complementary strand in a small region of space that crosses the outgoing strand pathway. In that region, which is shifted by two bases in the 3′ direction with respect to the heteroduplex melting bubble, five successive nucleotides of the complementary strand were found within or below pairing interaction distance from corresponding outgoing strand nucleotides all along the three 100-ns production trajectories (Supplementary Figures SI-9(B-E), SI-10(g)). Accordingly, transient or more stable pairing interactions spontaneously formed during the MD simulations between bases of the complementary and the outgoing strands: up to three simultaneous stable pairings were observed in one of the simulations, comprising two Hoogsteen pairings (49) and a triple-base pairing involving one base of each strand in a R-type geometry (50) (Figure 4). Interactions between one base of the outgoing strand and one phosphate of the complementary strand could also be observed in all four independent simulations. In addition, in a simulation with one Watson–Crick and one Hoogsteen pre-formed pairing interactions between the complementary and the outgoing strands, the Hoogsteen pairing remained stable during the whole 100-ns simulation while the less stable pre-formed Watson–Crick interaction was transferred during the simulation to a new nearby Watson–Crick base pair, which itself remained stable during the last 60 ns of the simulation (Supplementary Figure SI-12). These observations testify to the possibility of reverse pairing exchange associated to ATP-induced perturbations in the filament. Interestingly, in addition to the GSI group separating the complementary strand from the outgoing strand in 5′ of the melted region, we observe that another bulky group GSII formed by the LexA-binding loop of monomer (*i*) and the L2 loop of (*i* + 2), both of them tightly interacting all along the simulation (Figure 3C), separate back the outgoing strand from the complementary strand in 3′ of the distorted region, thus restoring the initial heteroduplex binding pattern.

## DISCUSSION

The present study examines the hypothesis that hydrolysis of ATP bound to a given monomer (*i*) results in this monomer modifying its binding geometry with monomer (*i* + 1) within a filament that otherwise presents an active geometry in which ATP is bound to each monomer. Our first significant result is that perturbations resulting from modifying a unique binding interface extend over almost two helical turns. This involves overall shape perturbations due to a kink in the filament axis between monomers (*i*) and (*i* + 1) and a complete reorganization of the filament internal space partitioning on the 3′ side of the hydrolysis spot due to the displacement of bulky structural motifs such as L2 or LexA-binding loops. Perturbations in terms of filament shape are characterized by large variations in the width of the filament groove, which ends up completely closed between monomers (*i*) and (*i* + 6). During the search for homology, the groove constitutes the gateway for non-specific binding of genomic DNA via interactions with the C-terminal domains (8,51), but also a binding site for partner proteins (52). Therefore variation in the filament groove width may modulate the access of both the DNA and the partner proteins to the filament in an ATP hydrolysis-controlled way.

In nucleofilament in the active form where no hydrolysis has taken place, the structures of bound ssDNA or dsDNA are remarkably stable in spite of important global stretching and unwinding deformations with respect to physiological B-DNA (53). The dsDNA structure in site I constitutes a mechanically optimal response to the overall deformation imposed by the filament helical characteristics (54–56), yet its stability mostly relies on stacking interactions involving hydrophobic groups of protein loops L1 and L2, inserted in large intercalation sites (21,57). The electrostatic potential generated by the whole filament also plays an important role in maintaining the complementary strand in site I in spite of few direct interactions with protein residues (53). It could be expected that distortion in the filament would result in large destabilization of the bound DNA.

A second important result of our study is therefore that the filament distortions remain compatible with the presence of up to three bound DNA strands. We find that perturbation in the geometry and binding interactions of the DNA in site I is confined over two successive base triplets initially bound to monomers (*i*) and (*i* + 1), while the DNA strand in site II modifies its course over four triplets from monomers (*i* – 1) to (*i* + 2). Uncoupling the perturbed region from the remaining DNA structure permits the DNA strands outside that region to conserve the structure and interaction they present in regular filaments. Because of this uncoupling, the ATP-hydrolysis independent initial 8-nucleotide recognition mechanism we proposed earlier (8), based on simulations at atomic resolution, remains possible in 5′ or in 3′ of the distorted region. Importantly, the filament perturbation in site I was not compatible with conserved Watson–Crick interactions in the kinked region, giving way to the formation of a 6-bp melting bubble. The presence of an outgoing strand in site II, that closely approached the complementary strand over a short 6-bp stretch overlapping the melting bubble, further contributed to destabilizing the heteroduplex melted region through the formation of transient or stable (reverse) pairing interactions with the complementary strand bases.

Destabilization of the heteroduplex and local reverse strand exchange are compatible with our recent experimental results (19) that showed strong destabilization of the RecA-induced strand exchange product in the presence of ATP hydrolysis. In this study, reverse strand exchange was demonstrated based on competition assays. The present model provides an interpretation at the atomic level for these observations. Its robustness, successfully challenged by molecular dynamics simulations, relies to a large extent on mechanical responses of the system to the large tension in the DNA strands and to the topological constraints inside the filament that strongly restrict the pathway followed by the strands.

Having shown that the hypothesis of individual monomer interface switching is structurally possible for all HR steps, we can discuss whether our simulations provide some hints regarding the possible modes of ATP hydrolysis propagation within the filament. Both the redistribution model and the cooperative propagation models involve a destabilization of specific monomer-monomer interfaces as a premise of either unbinding (here for (*i*),(*i* + 1)) or interface switching ((*i* − 1),(*i*) or (*i* + 1),(*i* + 2)). We did not observe any sign of such destabilization (Supplementary Figure SI-6). We note however that our simulations do not permit us to draw conclusions regarding the redistribution and cooperative propagation models since the simulation time is much shorter than the characteristic time for ATP hydrolysis (100 ns versus half a second) (29). Possible cooperation may be mediated by DNA unbinding from site I: our simulations show that when ATP of monomer (*i*) is hydrolyzed, that monomer loses most of the interactions that maintained the DNA in site I prior to hydrolysis. DNA unbinding from site I in consecutive monomers may favor an overall compressed form in the filament. However, if neighboring monomers retain an ATP-binding geometry, the DNA should easily recover its binding with site I after monomer (*i*) has regained its initial conformation at the end of a hydrolysis cycle, since the distorted DNA region is uncoupled from the rest of the bound bases. Local unbinding/rebinding in site I may facilitate gap elimination or sequence phasing reorganization recently observed in RecA filaments where ATP was hydrolyzed (58).

If our simulations cannot permit us to make conclusions about hydrolysis propagation to adjacent monomers, they strongly argue in favor of possible cooperation between distant monomers, across what initially was the filament groove. Cox *et al.* suggested that cooperation between monomers separated by five monomers might support their proposed hydrolysis wave model (41), but such cooperation does not occur in filaments in which all interfaces are in the ATP binding geometry. Here, we show that direct and stable interactions spontaneously appear between monomers (*i*) and (*i* +6) as a consequence of ATP hydrolysis in monomer (*i*), provided that hydrolysis is accompanied by local switch of the binding geometry. Along with the range of internal filament reorganization, which exactly covers six monomers, these interactions may establish the conditions for a cooperative mode of hydrolysis to develop along the filament, with a periodicity of six monomers (one helix turn). Indeed, our previous modeling showed that when repeated with a six-monomer periodicity, the global perturbations in the filament structure, that span two filament turns, nicely overlap within a left-handed superhelix (represented in Supplementary Figure SI-13) (24). The present results indicate that such superhelix may accommodate up to three DNA strands. Our models, however, do not provide any clear explanation for the periodic mode being observed only when two DNA strands are bound to site I. We speculate that the difference could depend on the cooperativity provided by the interactions between the tightly bound initiating strand and the complementary strand since ssDNA can be extended and twisted much more readily than dsDNA.

In the hydrolysis wave model, hydrolysis propagates every 0.5 s to the neighboring set of monomers distributed with a spatial periodicity of 6 monomers. How the melting bubbles in the heteroduplex DNA, which extend over two monomers, may progress from one hydrolysis event to the next one needs to be examined in future work. Remarkably, the changes in space partitioning within the three-stranded filament evoke a weaving machinery, where the DNA strands are alternatively brought in close proximity and separated by bulky protein regions (Figure 3C). Local reverse base pairing exchange may propagate along with the progression of the hydrolysis wave and destabilize the already formed heteroduplex, leading to full reverse strand exchange. Alternatively, the base pairs that transiently formed via reverse pairing exchange may break and return to their initial state. The possibility that either event occurs, or that they both occur with a given probability, remains an open question.

Finally, it will be interesting to determine whether the ATP hydrolysis pathway only involves one monomer that independently changes its binding mode or if it necessitates the concerted movement of several adjacent monomers, as simulated for cyclic ATPases such as the homohexameric transcription termination factor Rho (59). In the case of cyclic ATPases, preservation of the ring closure requires the changes in binding modes to be concerted and, indeed, single molecule experiments revealed high degree of cooperativity in such systems (60–64). This is not necessarily the case for open helical systems such as the RecA filament and the question merits to be further explored.

## CONCLUSION

We believe that the present results unveil essential aspects of the dynamics of ATP-driven HR mechanism. They provide atomic level interpretation for recent observations of ATP-induced destabilization of the product of strand exchange and the instability of dsDNA binding to site I. Given the convergence in cofactor-dependent filament characteristics between the eukaryotic and prokaryotic recombinases (65), we can expect this mechanism to have a universal character, although eukaryotic recombinases may exhibit some functionally important differences. These proteins notably are weak ATPases; however they are assisted by auxiliary proteins that increase the rate of ATP turnover (66). Our results strongly appeal to more extensive studies of the structural role of ATP hydrolysis.

## DATA AVAILABILITY

Coordinates of filament structures with mixed ATP/ADP interfaces and bound DNA are available from the Model Archive database with codes ma-eaaa9, ma-900pk and ma-l1kfl.

## Supplementary Material

gkz667_Supplemental_Files
