# Supplementary material for: Weaving DNA strands: structural insight on ATP hydrolysis in RecA-induced homologous recombination
Source: Nucleic Acids Res. 2019 Aug 2;47(15):7798–808. doi: 10.1093/nar/gkz667 (PMC6735932; doi:10.1093/nar/gkz667)
Supplement: gkz667_Supplemental_Files [file gkz667_supplemental_files.zip › SI-I.pdf]

Weaving DNA strands: structural insight on ATP hydrolysis in RecA-induced homologous recombination

B. Boyer, C. Danilowicz, M. Prentiss, and C. Prévost

Supplementary information SI-I

List of figures :

Figure SI-1. Details of protein-DNA interactions within the twelve-monomer RecA nucleofilament with one central ADP-type interface.

Figure SI-2. Time evolution of the groove entrance width.

Figure SI-3. Time evolution of intra-filament distances.

Figure SI-4. Root mean square deviation of loops L1 and L2 of individual RecA monomers.

Figure SI-5. Root mean square fluctuation.

Figure SI-6. Stability of the monomer/monomer interfaces.

Figure SI-7. Root mean square deviation.

Figure SI-8. Superposed cartoon representations of DNA strands bound to the twelve-monomer RecA nucleofilament.

Figure SI-9. Inter-strand phosphate-phosphate distances.

Figure SI-10. Stability of a three-stranded filament with reversed Watson-Crick pairing.

Figure SI-11. Rearrangement of the internal space partitioning within a three-stranded filament.

Figure SI-12. Stability of reversed Watson-Crick and Hoogsteen base pairing.

Figure SI-13. Superhelical structure resulting from a periodic distribution of ADP-type interfaces every six monomers.

Supplementary information SI-II

Movie SI-II (SI-II.mpeg, separate file). 3-D surface view of a twelve-monomer RecA nucleofilament with one central ADP-type interface and a DNA strand bound in site I. The protein is in surface representation in white (core), The protein filament is represented in white, with the C-terminal domains in pink and the N-terminal domains in magenta. The DNA strand is in orange.

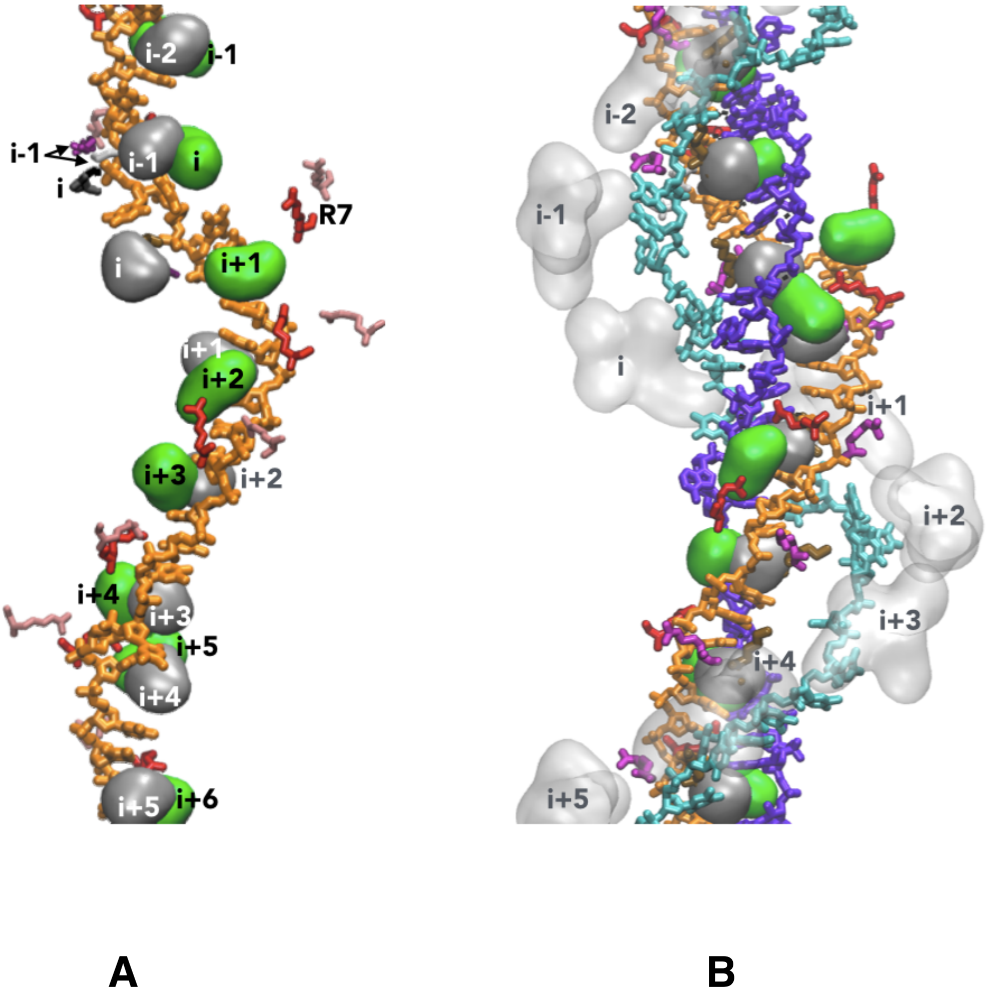

Figure SI-1. **Details of protein-DNA interactions within the twelve-monomer RecA nucleofilament with one central ADP-type interface.** The ADP-type interface is situated between monomers R6 and R7. (A) Protein-DNA interactions between RecA amino-acids and a single-stranded DNA in site I; the DNA strand and amino-acids involved in polar interactions (hydrogen-bonds or salt bridges) are represented in licorice, with color codes as follows: DNA strand (orange) Arg196 (magenta), (*i-1*):Arg176 (pink), Arg169 (red), (*i*):Lys198 (marron), (*i-1*):Met197 (white), (*i*):Asn213 (black); Ile199 (silver) and Met164 (green) that form hydrophobic contacts with the DNA bases are in surface representation; monomers are labelled from (*i-1*) to (*i+6*); distortions in the kinked region involve contact separation between (*i*):199 and (*i+1*):164, removal of (*i+1*):169 and (*i+1*):176 from their contacts in site I, creation of new stabilizing contacts with (*i*):Arg198 and, at the 5' extremity, with (*i-1*):Met197 and (*i*):Asn213. (B) Protein-DNA interactions in the three-stranded complex; the color code for the protein amino acids is the same as in A but all Arg196 and Lys198 residues are represented; outside the distorted regions, Lys198 residues are equidistant from the phosphate groups of the three strands; from monomers (*i+1*) to (*i+3*) Lys198 gets close to phosphate groups of one of the strands (incoming strand for (*i+1*), outgoing strand for (*i+2*) and (*i+3*)) at the expense of the other two; the complementary and outgoing DNA strands are respectively represented in blue and cyan; clusters of acidic residues Lys245, Arg226, Arg227 and Arg243, that bind phosphodiester groups of the outgoing strand in site II, are represented with white transparent surface and labelled with the monomer index.

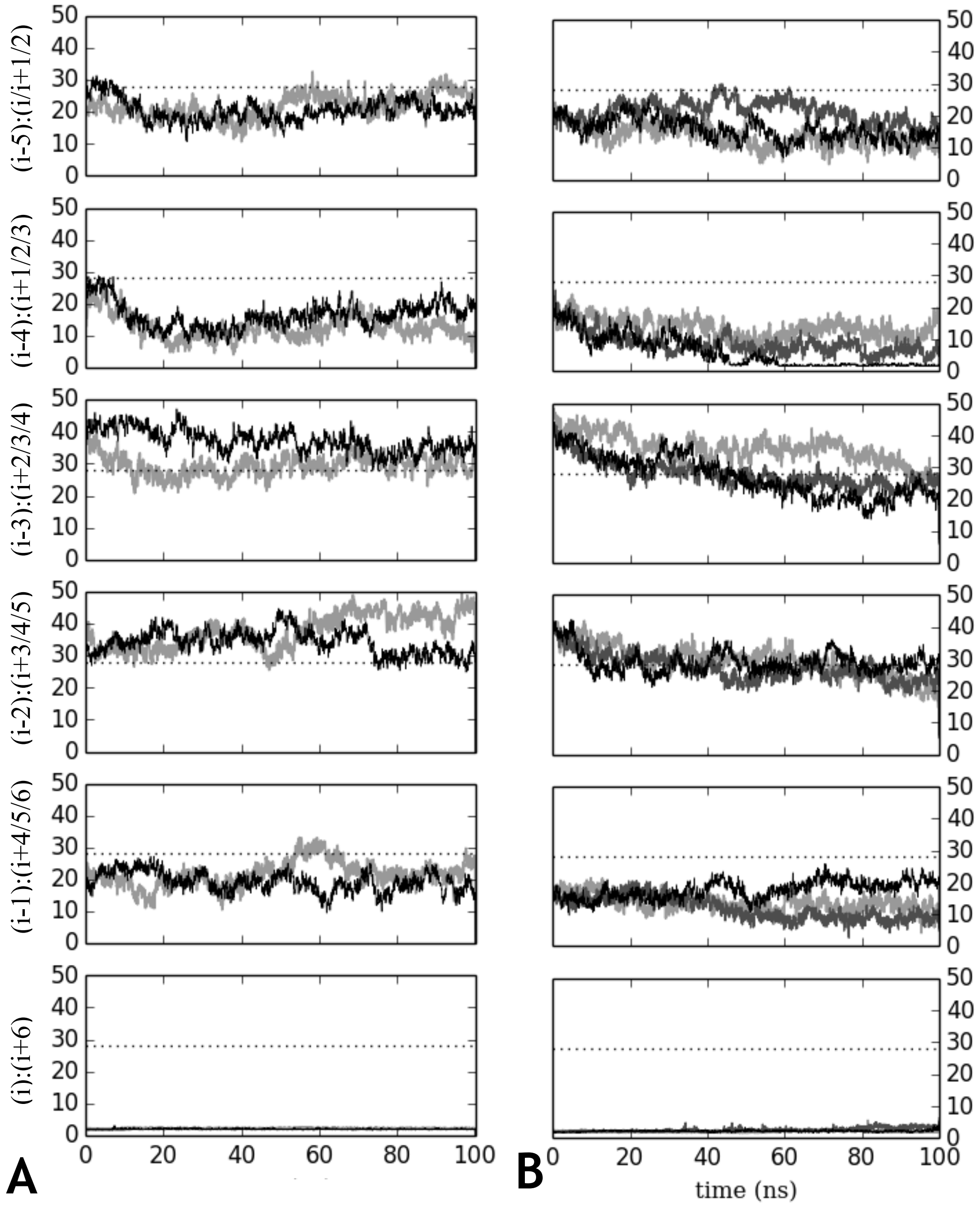

Figure SI-2. **Time evolution of the groove entrance width** (in Å) for the RecA nucleofilament with one central interface in the ADP-type, measured at the level of monomers (*i-5*) to (*i*) during MD simulations with (A) one (light grey), or two (black) DNA strands and (B) three DNA strands, during three independent simulations. The black line in B in figures SI-2 to SI-4 and SI-7 corresponds to the simulation showing reverse pairing exchange (Figure 4, main text). The groove entrance width is taken as the shortest distance across the groove between the C-terminal domain of reference monomer (*k*) and the N-terminal domains of the three closest monomers across the groove (monomers (*k+5*), (*k+6*), (*k+7*)). The horizontal dotted line represents the width of the groove entrance in a regular filament built from the crystal structure with PDB code 3CMW (average width value : 28 Å).

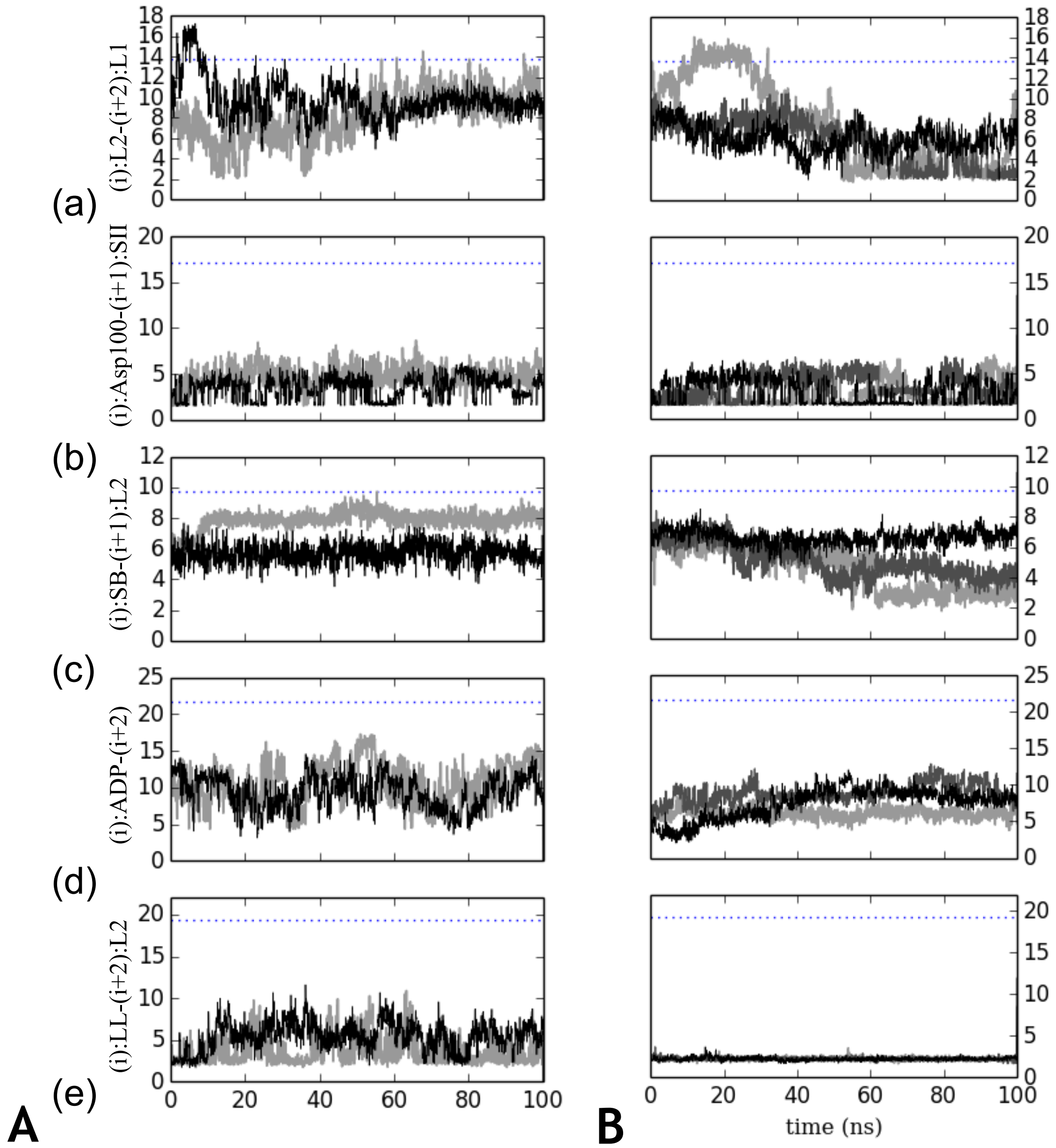

Figure SI-3. **Time evolution of intra-filament distances** (in Å) between groups of atoms within a RecA nucleofilament with one central interface in the ADP-type, containing (A) one (grey) or two (black) DNA strands, (B) three DNA strands in three independent simulations. Values for regular active filaments are represented with blue horizontal dotted lines. In the vertical labels, LL represents a LexA-binding loop (residues 225 to 245), SB the two residues Lys226 and Glu206 forming a salt-bridge that anchors loop L2 to the protein core, and SII represents positively charged residues Lys245 and Arg243 in site II. (a) shortest distance between residue Ile199 of (*i*):L2 and residues His163, Met164 of (*i+2*):L1; in the regular filament, the distance is 13.7 Å; (b) shortest distance between residue Asp100 of monomer (*i*) and the SII group of monomer (*i+1*); the corresponding distance in regular filaments is 17.1 Å;(c) closest distance between the SB group of monomer (*i*) and residues Met202, Phe203 of monomer (*i+1*); in regular filaments, this distance is 9.7 Å, close to the value observed in the filament with a single strand; the presence of a second, and more markedly of a third strand can drive the (*i+1*):L2 loop to closely approach the 206-226 salt bridge; (d) shortest distance between (*i*):ADP and the monomer (*i+2*); in the regular filament, the distance between (*i*):ATP and (*i+2*) is 21.6 Å; (e) shortest distance between the LexA-binding loop of monomer (*i*) and residues Met202 and Phe203 of (*i+2*):L2 loop; the corresponding distance in the regular filament is 19.3 Å; when a third strand is present in site II (B-e) the two regions are in strong contact and neatly partition the filament groove.

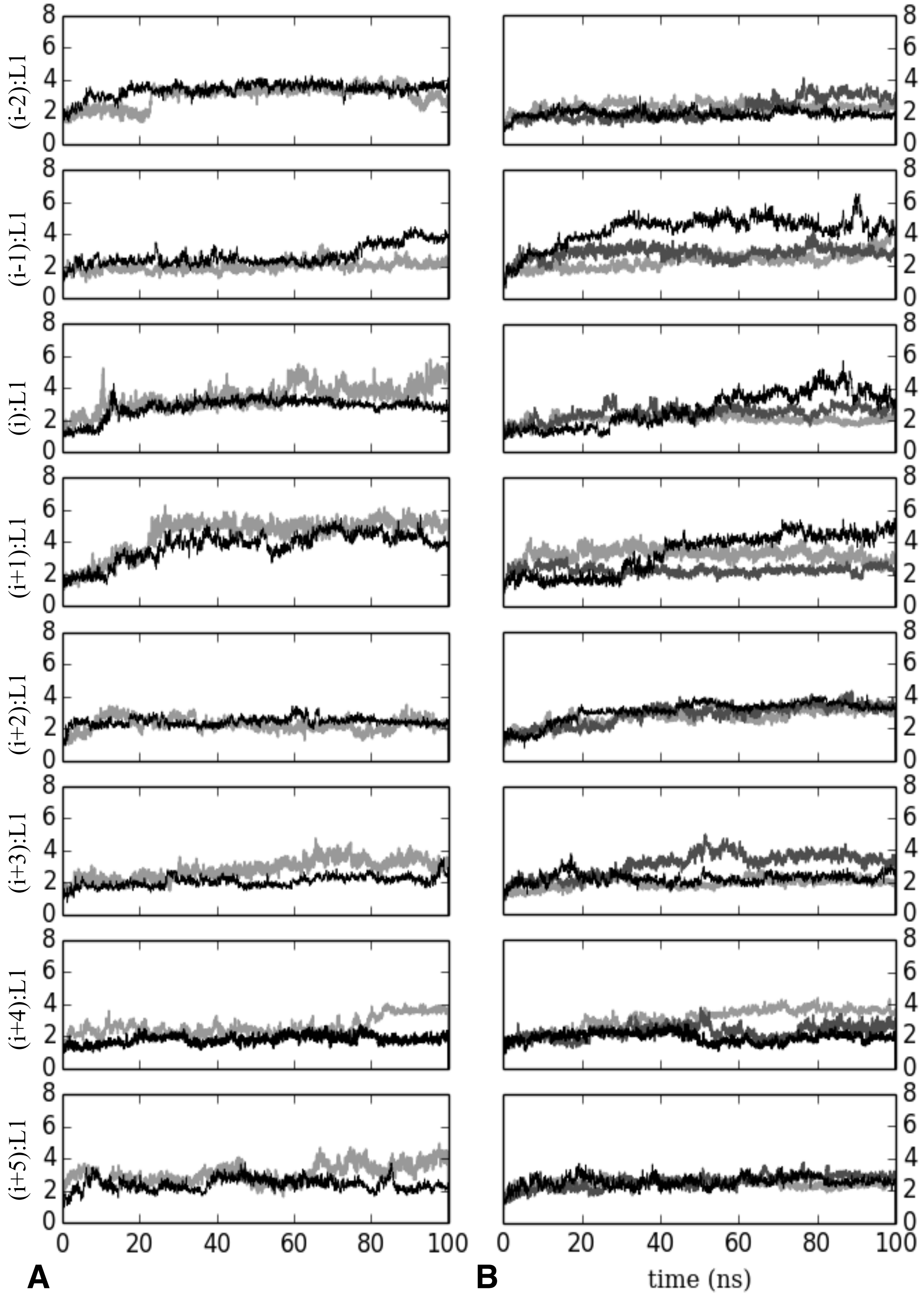
(i-2):L1
(i-1):L1
(i):L1
(i+1):L1
(i+2):L1
(i+3):L1
(i+4):L1
(i+5):L1
0 2 4 6 8
0 20 40 60 80 100
A
B
time (ns)

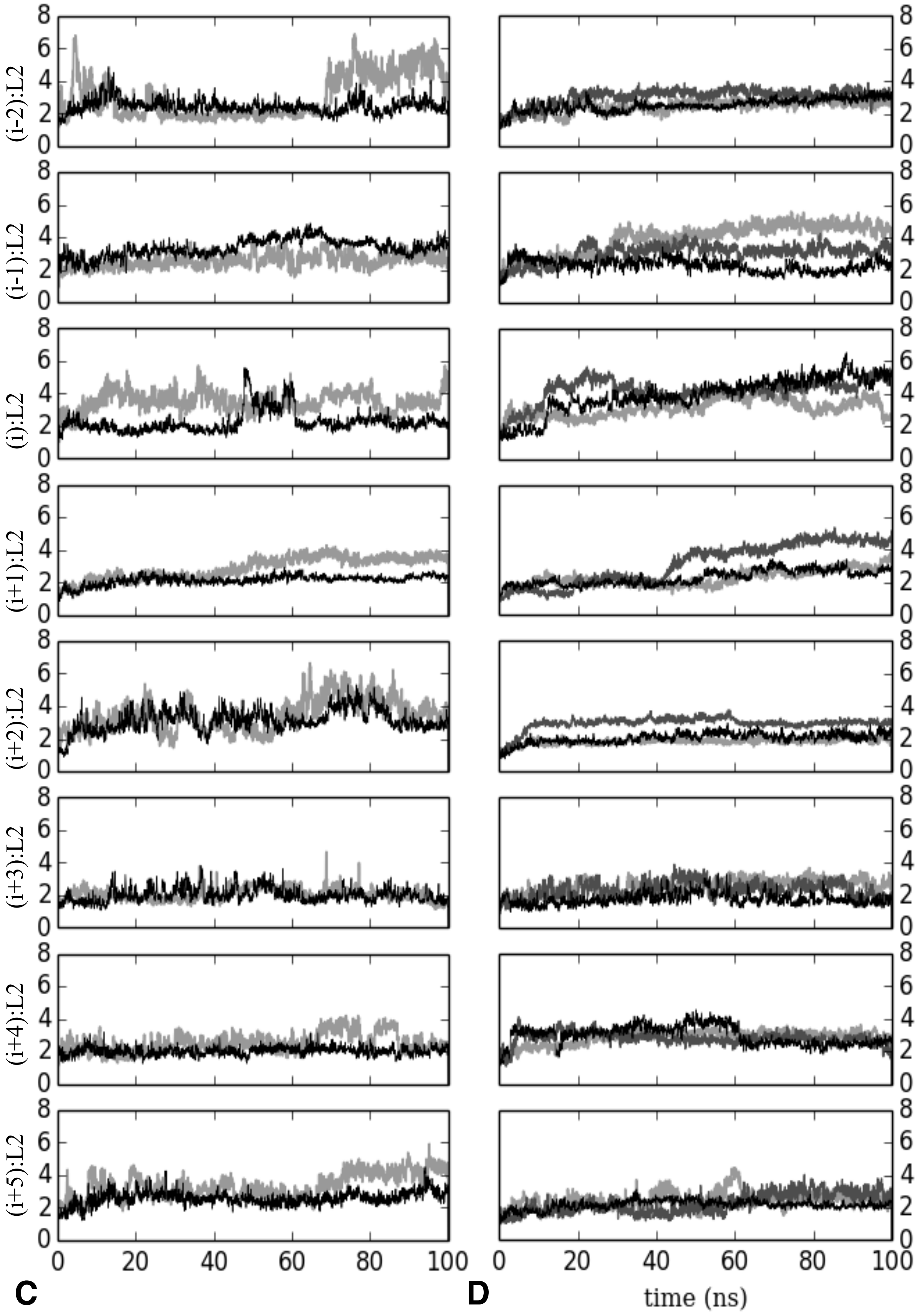

Figure SI-4. **Root mean square deviation** (RMSD, in Å) **of loops L1** (A, B) **and L2** (C, D) **of individual RecA monomers** during 100 ns of molecular dynamics simulation of the nucleofilament with central ADP-like interface with (A, C) one (light grey) or two (grey) DNA strands and (B, D) three independent MD runs on the three-stranded complex. The RMSD values have been taken on the C_α atoms.

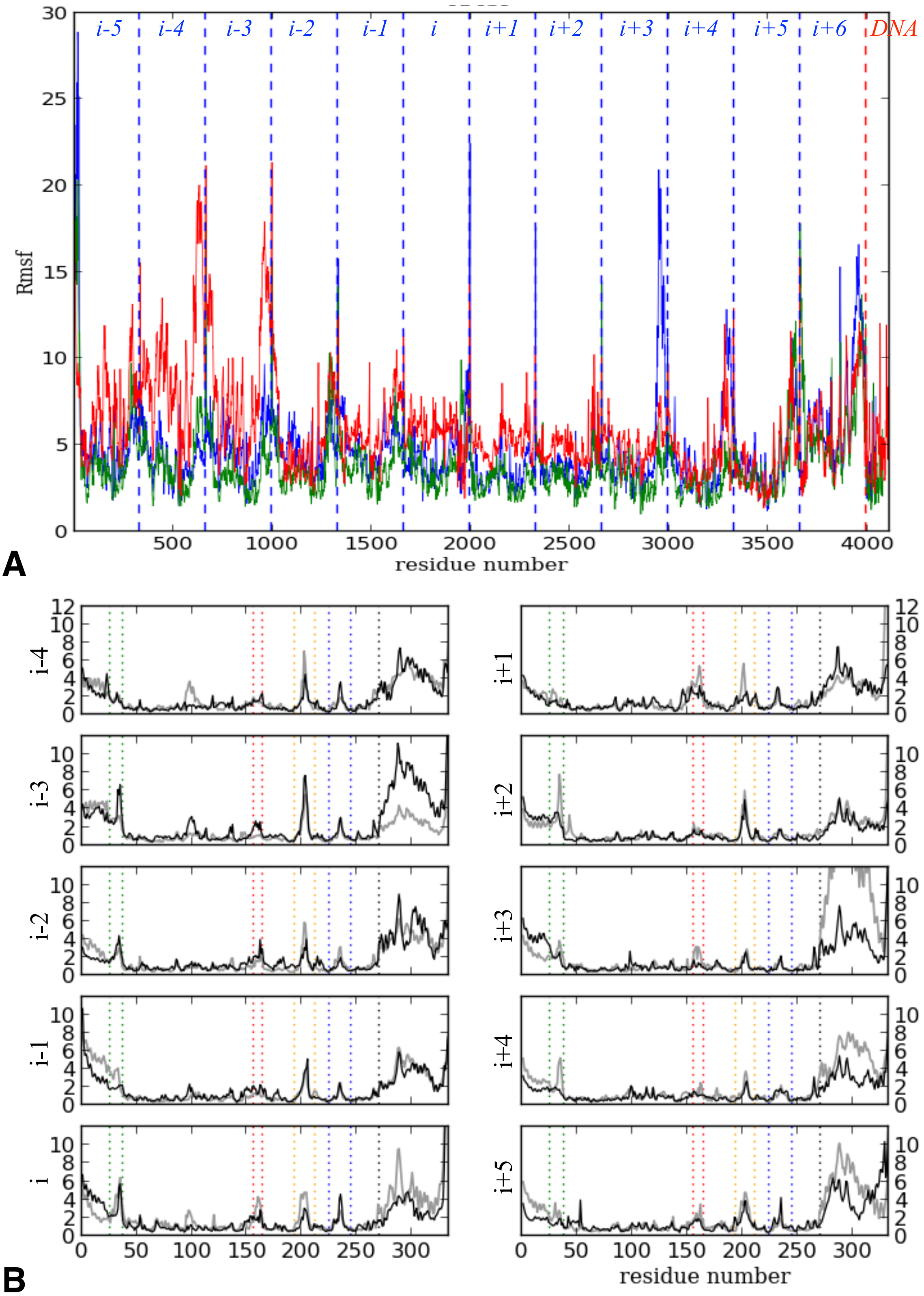
A
B
Rmsf
residue number
i-5
i-4
i-3
i-2
i-1
i
i+1
i+2
i+3
i+4
i+5
i+6
DNA
i-4
i-3
i-2
i-1
i
i+1
i+2
i+3
i+4
i+5
residue number

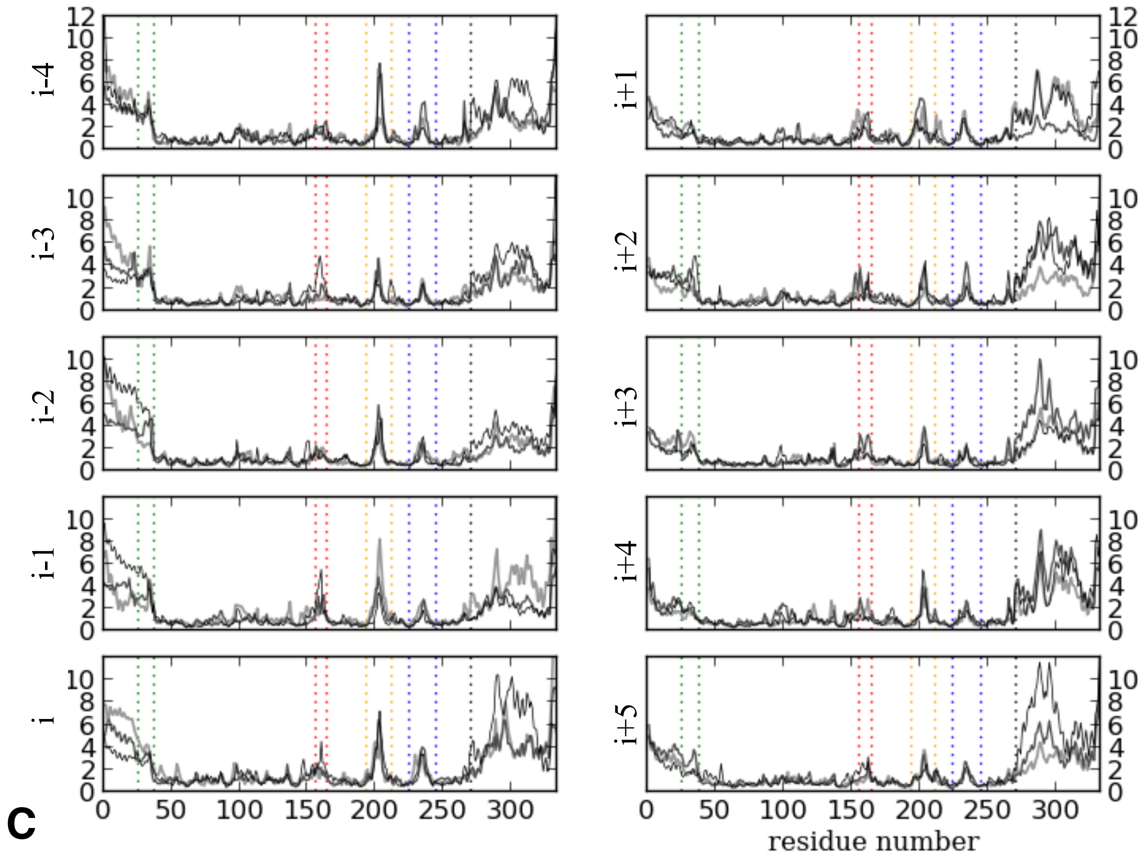

Figure SI-5. **Root mean square fluctuation.** (A) Root mean square fluctuation (RMSF) of the RecA nucleofilament with central ADP-type interface during 100 ns molecular dynamics simulation with one (blue), two (green) and three (red) DNA strands; residues are numbers from 1 to 4092 (333 residues for each monomer (*i-4*) to (*i+5*), separated by blue vertical broken lines and 36 residues for each of the three DNA strands, with a red vertical broken line separating protein from nucleic acid residues); (B, C) RMSF for each individual RecA monomer after superposition of the MD frames restricted to that monomer; for each monomer, colored dotted lines delineate the N-terminal helix and linker (green), the L1 loop (red), L2 loop (orange), LexA-binding loop (blue) and the C-terminal domain (black); the values represented in (B) concern the systems with one (light grey) and two (black) DNA strands; in (C) three grey shades represent three independent MD runs of the three-stranded complex.

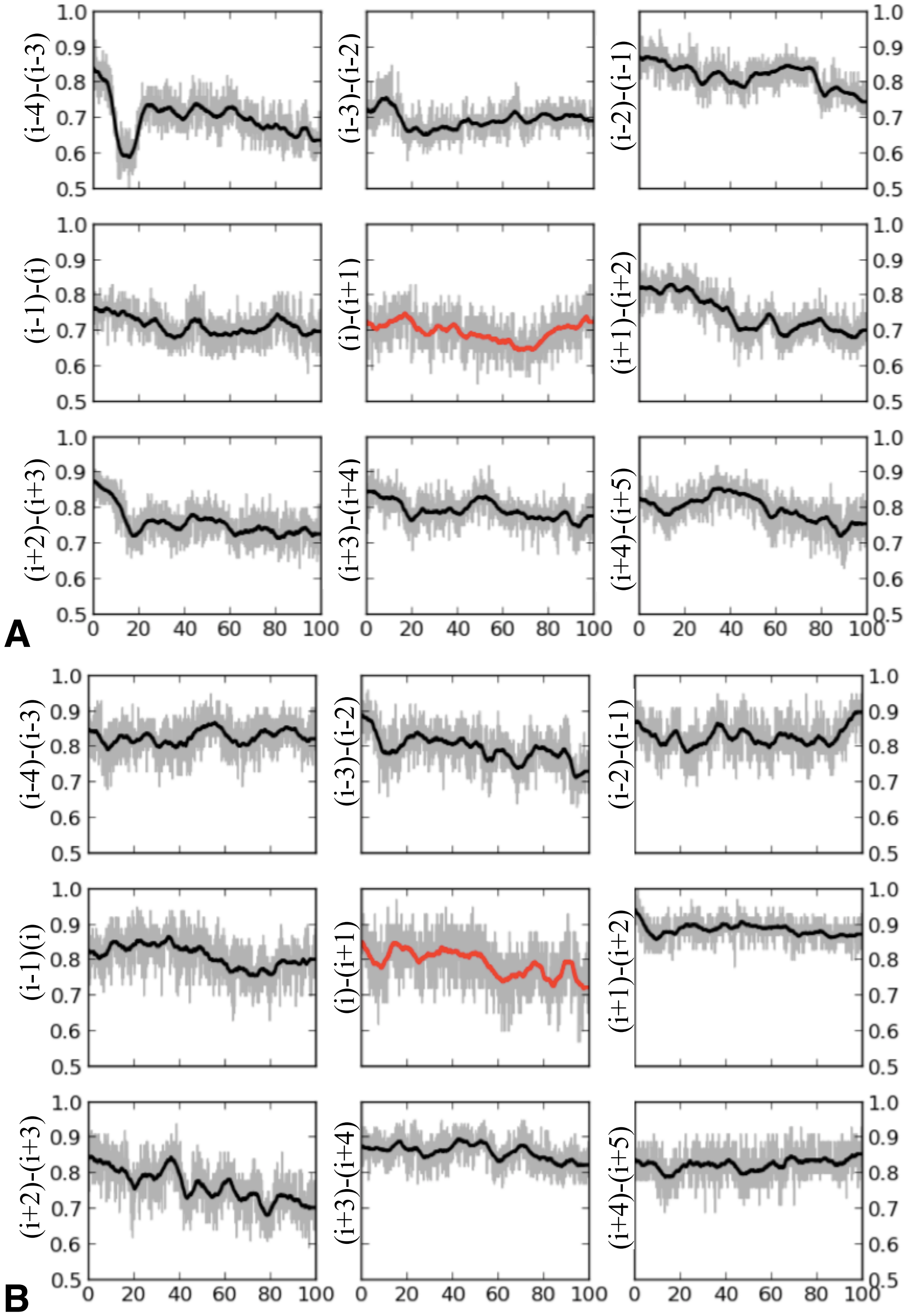

Figure SI-6. **Stability of the monomer/monomer interfaces.** Time evolution of F_{NAT}, the fraction of conserved pair contacts between rigid regions of consecutive RecA monomers during 100 ns molecular dynamics simulation of a nucleofilament with central ADP-like interface and (A) two bound DNA strands; (B) three bound DNA strands. The interface is considered to be conserved if the f_{NAT} value exceeds 0.5 (Mendez et al., Proteins 2005, 60:150-169). F_{NAT} values are calculated with respect to the starting structure, i.e. the structure with PDB code 3CMX for all interfaces except the (i),(i+6) ones where the reference is the structure with PDB code 2REB (red curve). Definition of the rigid region excludes the L1 and L2 flexible loops (residues 156-165 and 194-212) and the N-terminal domain and linker (1-37) (see Boyer *et al.*, PlosOne 2015, 10, e0116414).

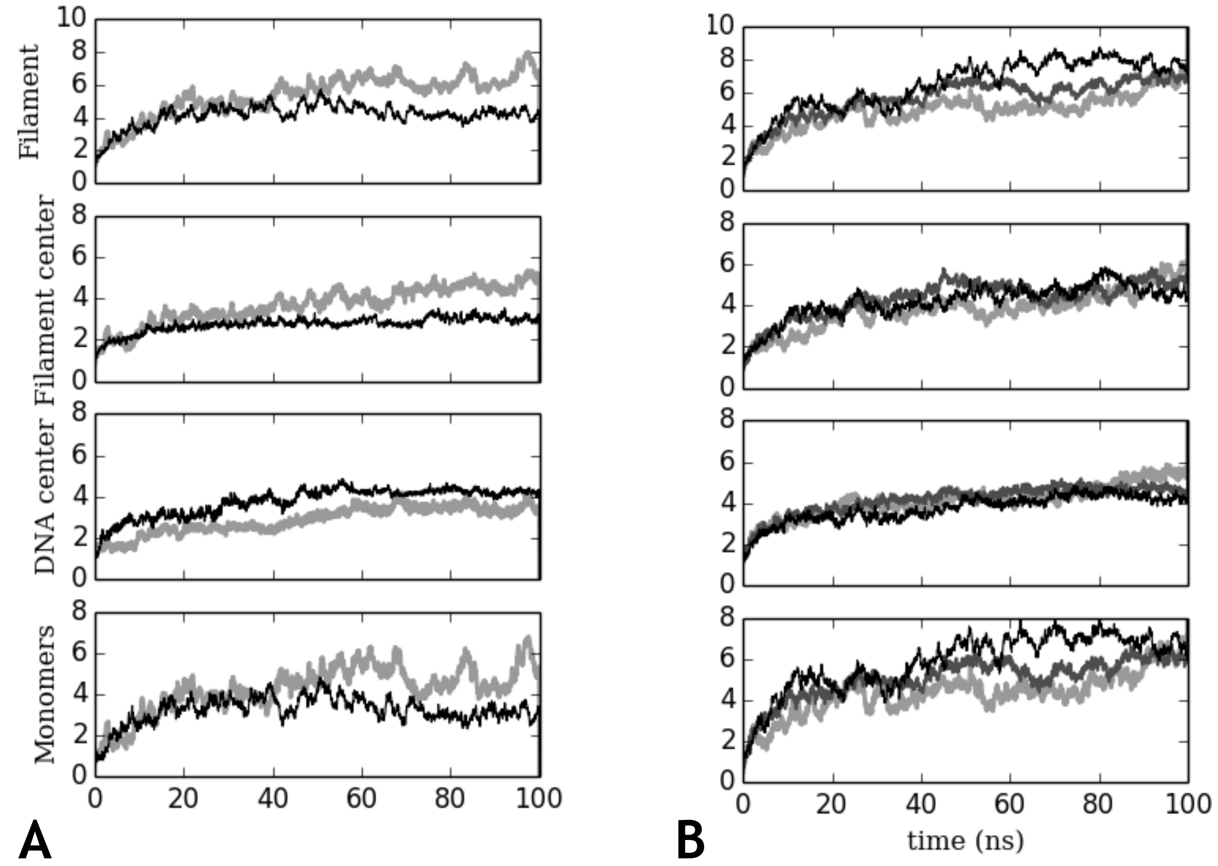

Figure SI-7 Root mean square deviation (RMSD) of selected regions of the RecA nucleofilament with central ADP-like interface during 100 ns of molecular dynamics simulation (A) with one (light grey) or two (black) DNA strands (B) with three DNA strands during three independent MD runs. From top to bottom, the selected regions are the whole protein filament, RecA monomers (*i-2*) to (*i+3*), DNA residues bound to RecA monomers (*i-2*) to (*i+3*) and the average RMSD value of individual monomers after superposition restricted to this monomer. This permits separating out the impact of global filament movements on the RMSD values. All RMSD values are taken on the C_α atoms (protein) and the P atoms (nucleic acid).

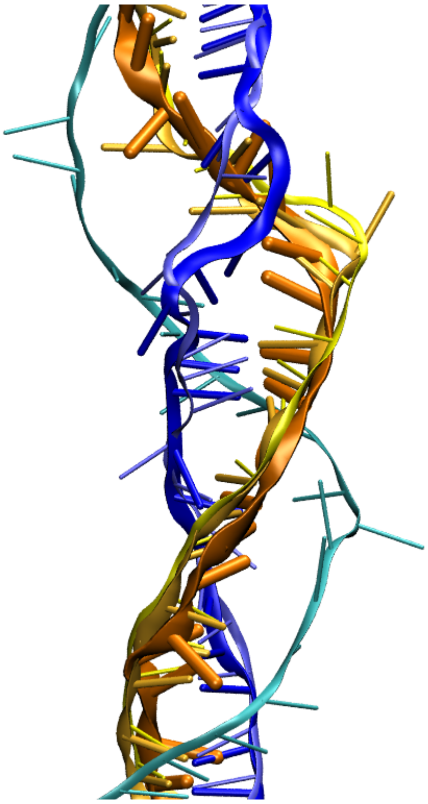

Figure SI-8. **Superposed cartoon representations of DNA strands bound to the dodecameric RecA nucleofilament** with one central ADP-type interface, when one (orange, large ribbon), two (light orange and blue, medium ribbons) and three (yellow, light blue and cyan, thin ribbons) DNA strands are bound. The snapshot structures have been taken at the end of the 100 ns simulations.

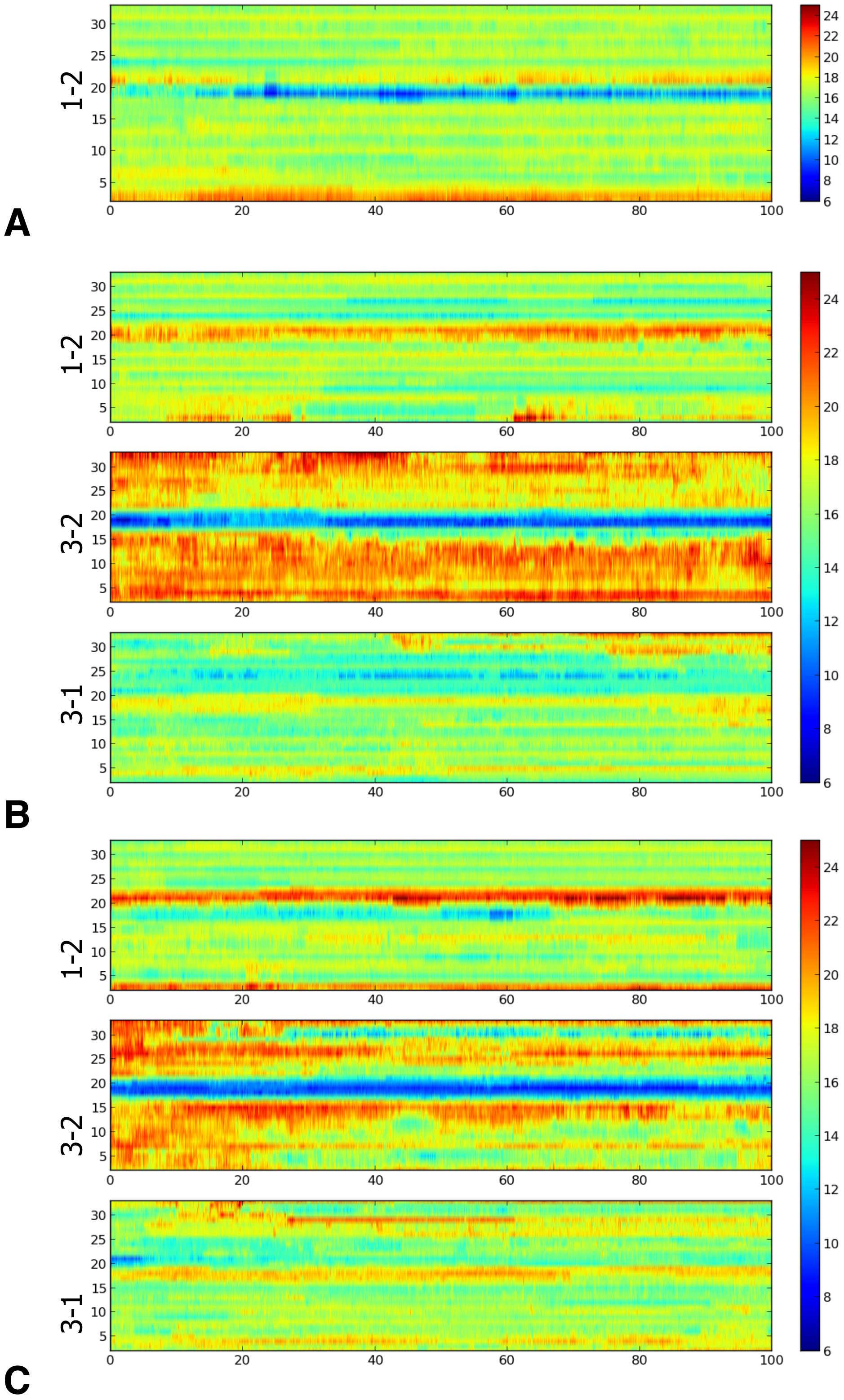
A
1-2
B
1-2
3-2
3-1
C
1-2
3-2
3-1
0
20
40
60
80
100
5
10
15
20
25
30
6
8
10
12
14
16
18
20
22
24

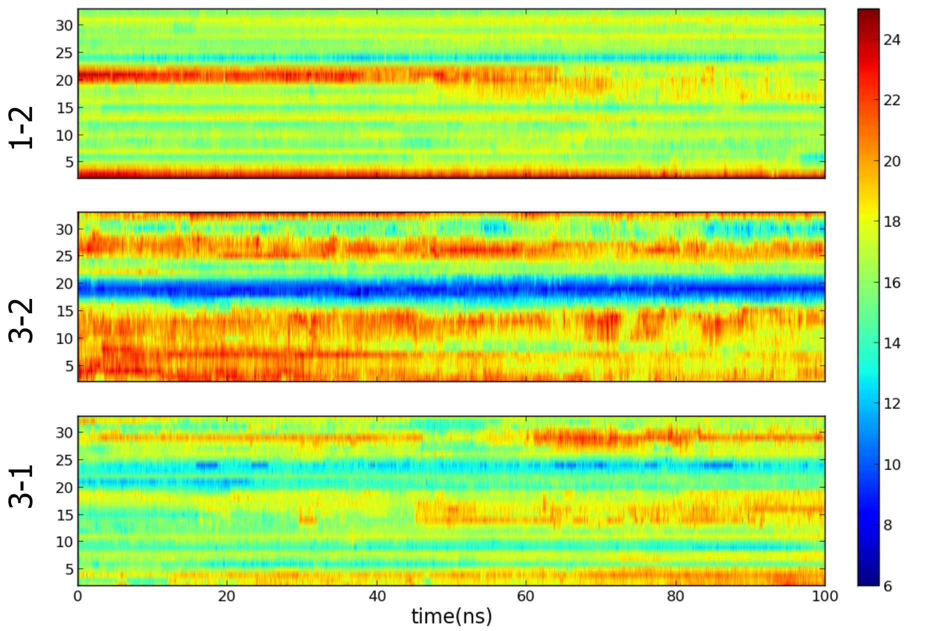

D

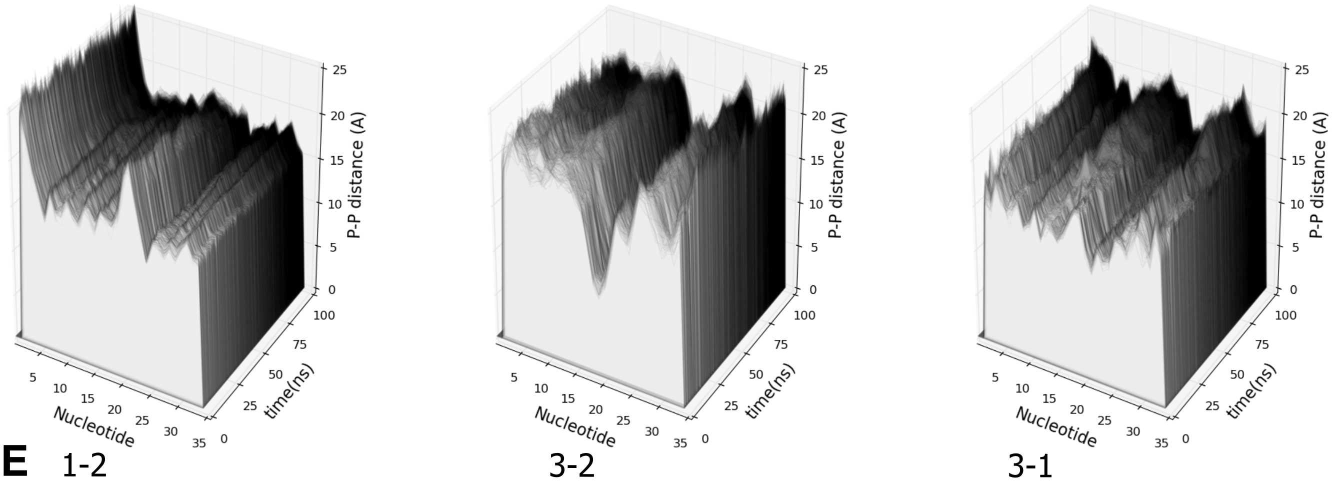

E

Figure SI-9. **Inter-strand phosphate-phosphate distances**. Time evolution, during 100 ns runs, of the shortest distance between the phosphate of thymine *y* in strand *j* (y-axis, from 1 to 36 in the 3'-5' direction) and all phosphates of strand *k* (*j*-*k* = 1-2, 3-2, 3-1), where '1' represents the first strand in site I, '2' the complementary strand in site I and '3' the outgoing strand in site II. The simulations were run on systems with (A) two, or (B-E) three DNA strands. The (A-D) plots are colored according to distance values ranging from 6 Å (blue) to 25 Å (red). Bar scales are represented on the right. Typical distances where Watson-Crick interactions can be observed are 16 to 18 Å. (E) Same data as (D) displayed in 3D representation.

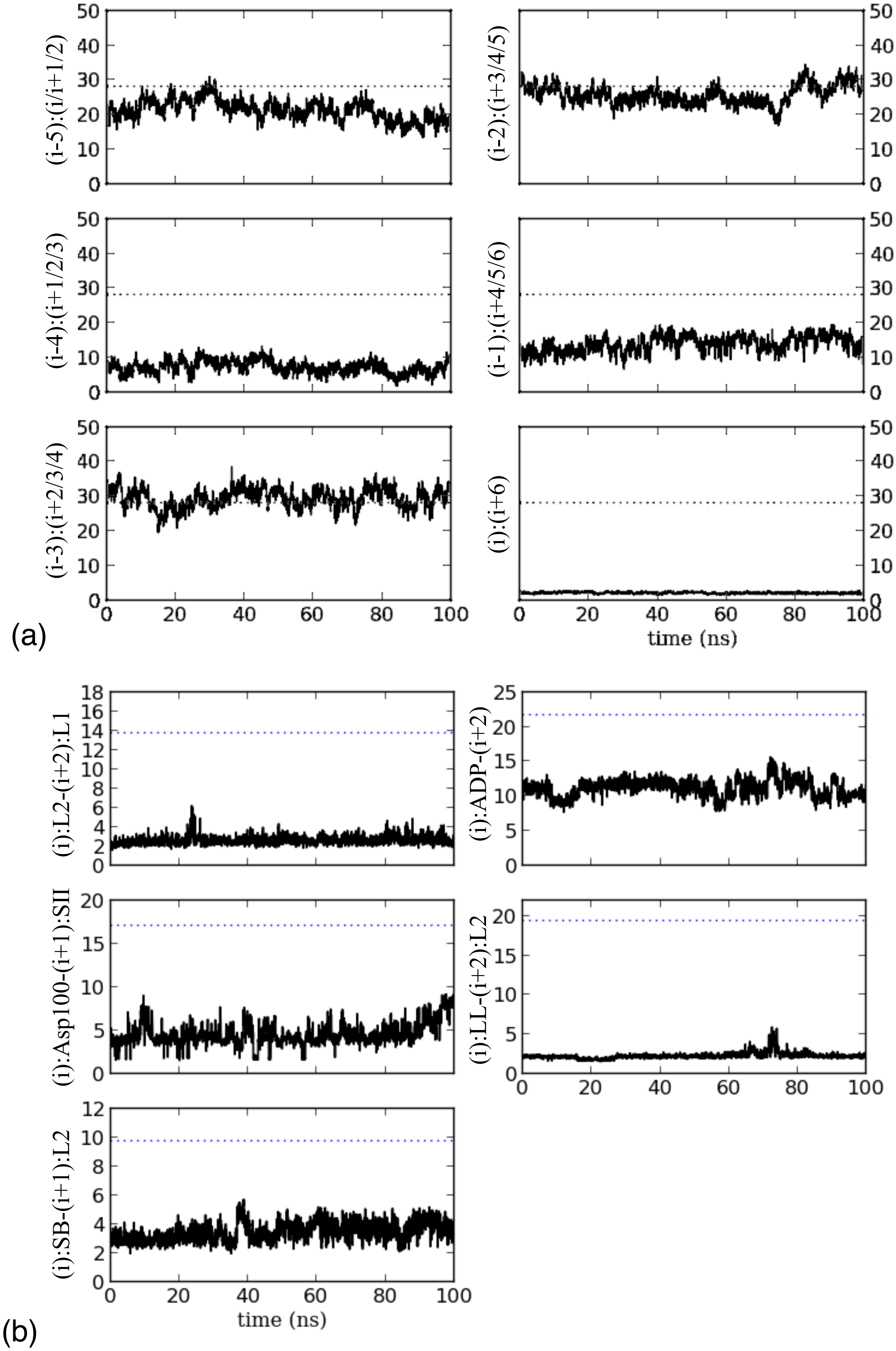
(i-5):(i/i+1/2)
(i-2):(i+3/4/5)
(i-4):(i+1/2/3)
(i-1):(i+4/5/6)
(i-3):(i+2/3/4)
(i):(i+6)
time (ns)
(a)
(i):L2-(i+2):L1
(i):ADP-(i+2)
(i):Asp100-(i+1):SII
(i):LL-(i+2):L2
(i):SB-(i+1):L2
time (ns)
(b)

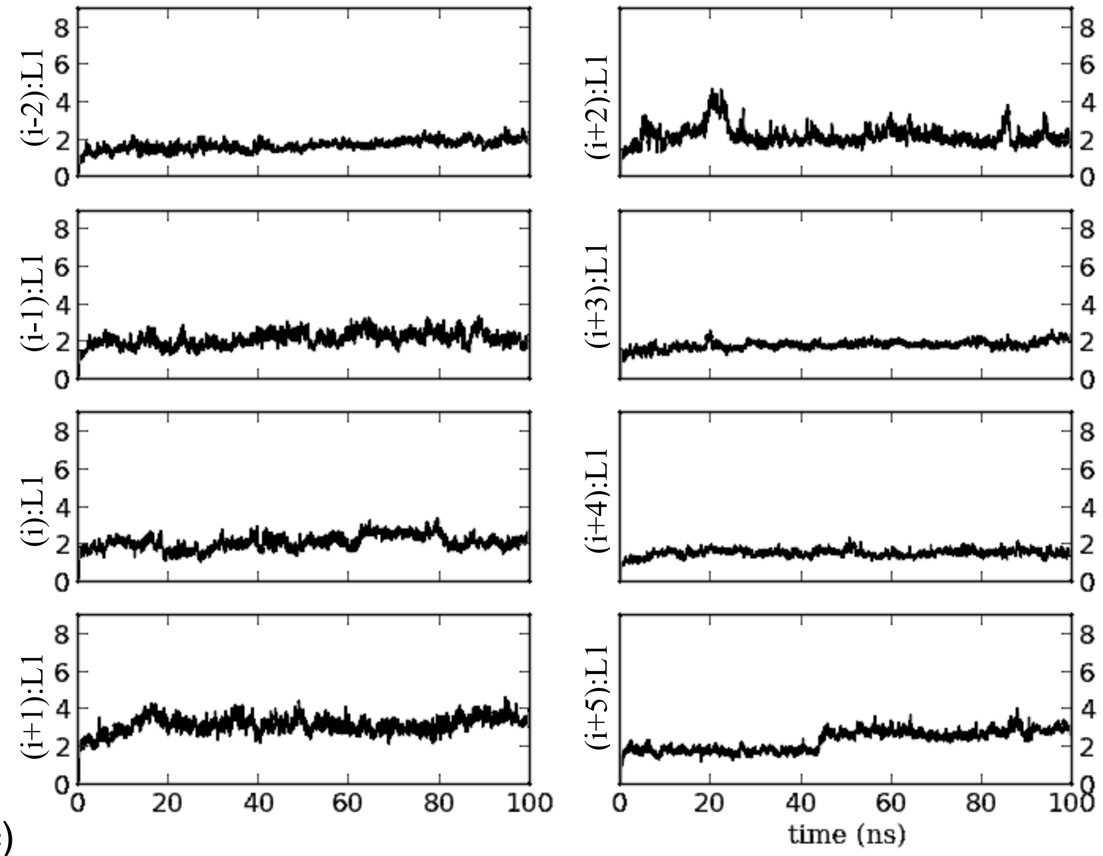
(i-2):L1
(i-1):L1
(i):L1
(i+1):L1
(i+2):L1
(i+3):L1
(i+4):L1
(i+5):L1
time (ns)

(c)

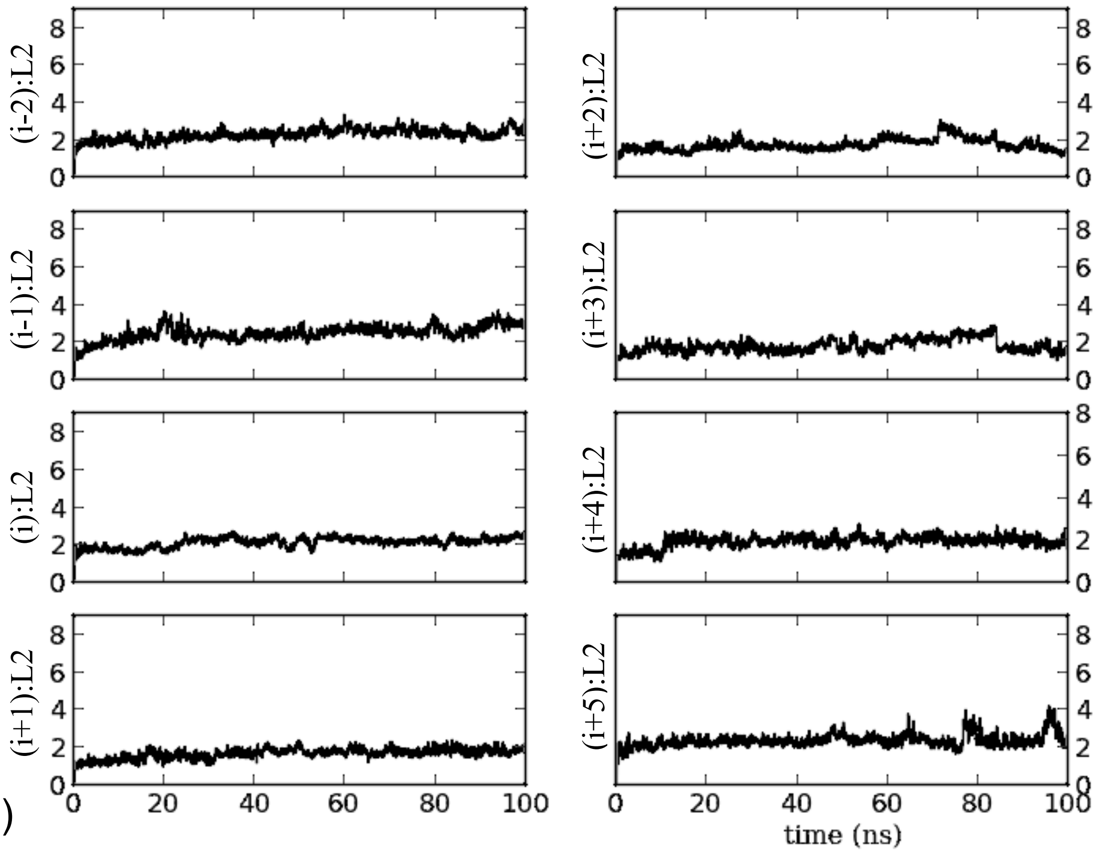
(i-2):L2
(i-1):L2
(i):L2
(i+1):L2
(i+2):L2
(i+3):L2
(i+4):L2
(i+5):L2
time (ns)

(d)

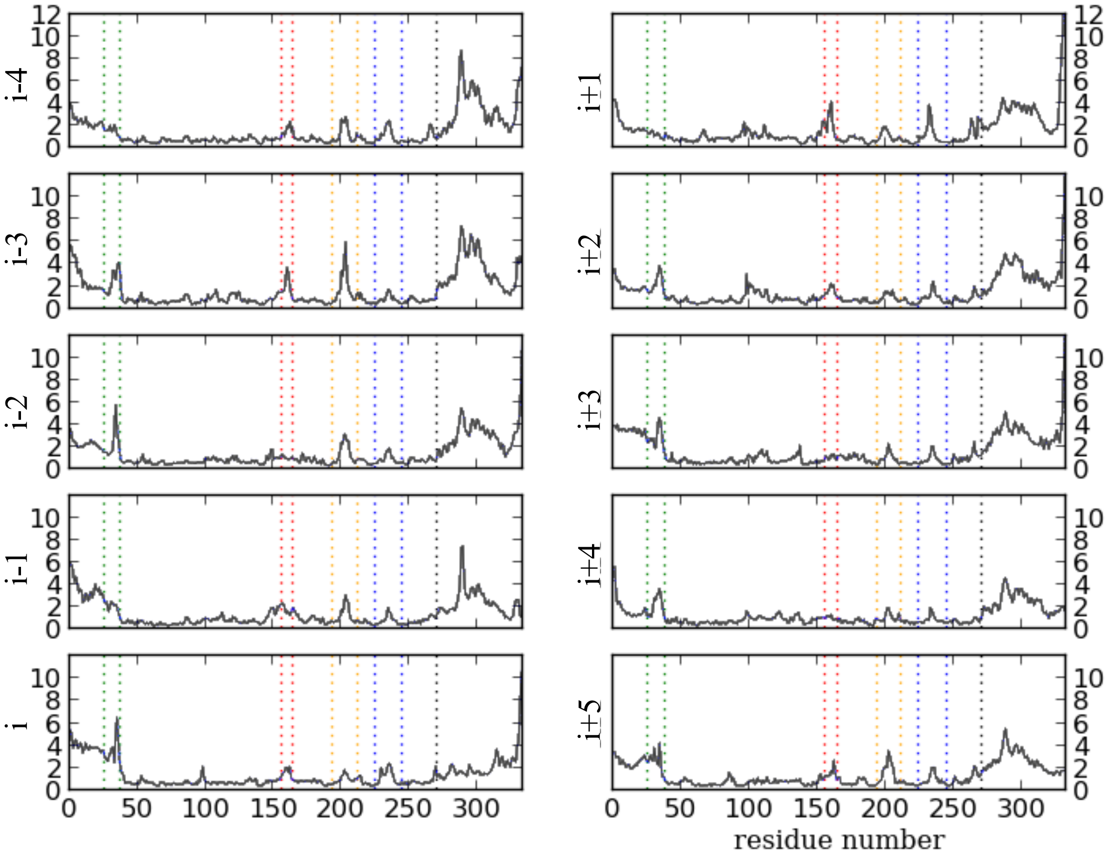
i-4
i-3
i-2
i-1
i
i+1
i+2
i+3
i+4
i+5
residue number

(e)

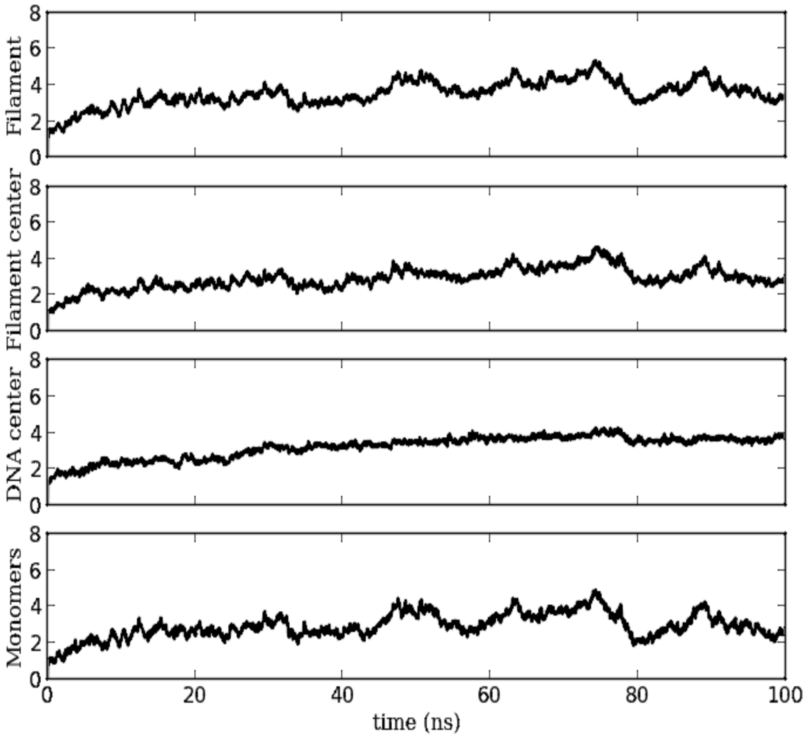
Filament
Filament center
DNA center
Monomers
time (ns)

(f)

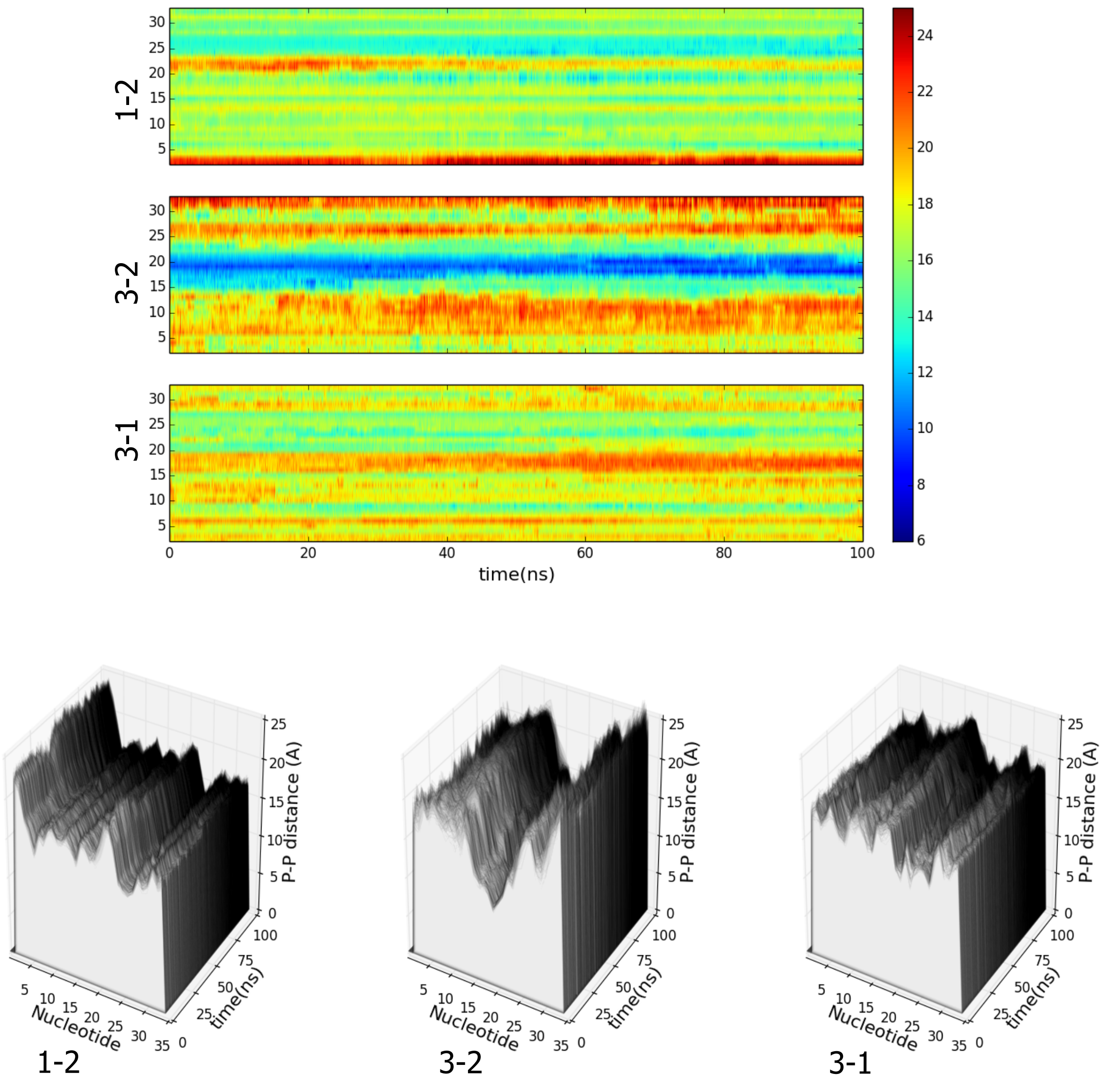

(g)

Figure SI-10. Stability of a three-stranded filament with reversed Watson-Crick pairing. Time evolution, during 100 ns MD simulation, of different values characterizing the geometry and fluctuations of a RecA nucleofilament with central ADP-like interface and three DNA strands, where the starting structure was constructed with a reversed Watson-Crick base pair in the distorted region; the plots (a) to (g) are given in the same order as figures SI-2 to SI-5, SI-7 and SI-9, with identical label and color code conventions; (a) groove entrance width; (b) internal distances between selected groups of atoms; (c,d) C_{α}-RMSD of L1 and L2 loops, respectively, with respect to their initial position in monomers (*i-2*) to (*i+5*); (e) RMSF for monomers (*i-4*) to (*i+5*); (f) RMSD of regions of the filament defined in Figure SI-7; (g) two representations of the evolution of the shortest inter-phosphate distance between strands 1 and 2, 3 and 2, 3 and 1 (see Figure SI-9).

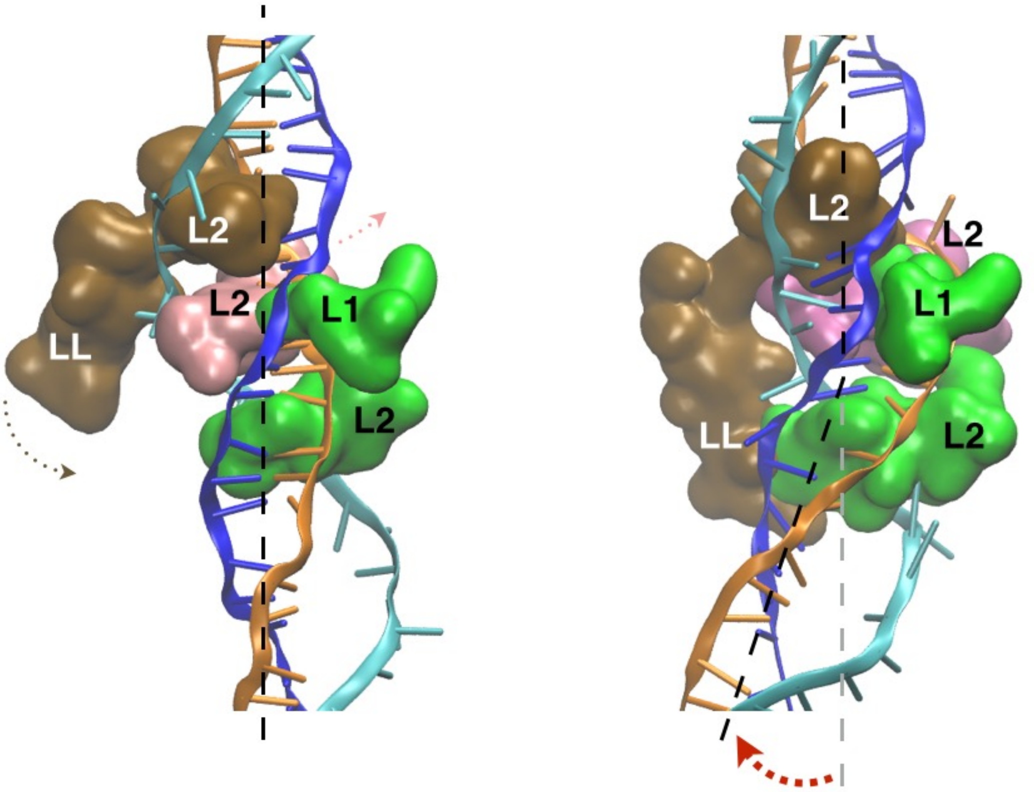

Figure SI-11. Rearrangement of the internal space partitioning within a three-stranded filament. The arrangement of bulky loops L2, L1 and a lexA-binding loop (LL) from three successive monomers ((*i*) in marron, (*i+1*) in pink, (*i+2*) in green) is shown before (left panel) and after (right panel) modification of the (*i*),(*i+1*) binding interface from a geometry favorable to ATP binding to a geometry favorable to ADP binding. The filament axis is represented with a black broken line. The changes in bulky loops relative position, as well as the change in axis orientation are represented with arrows.

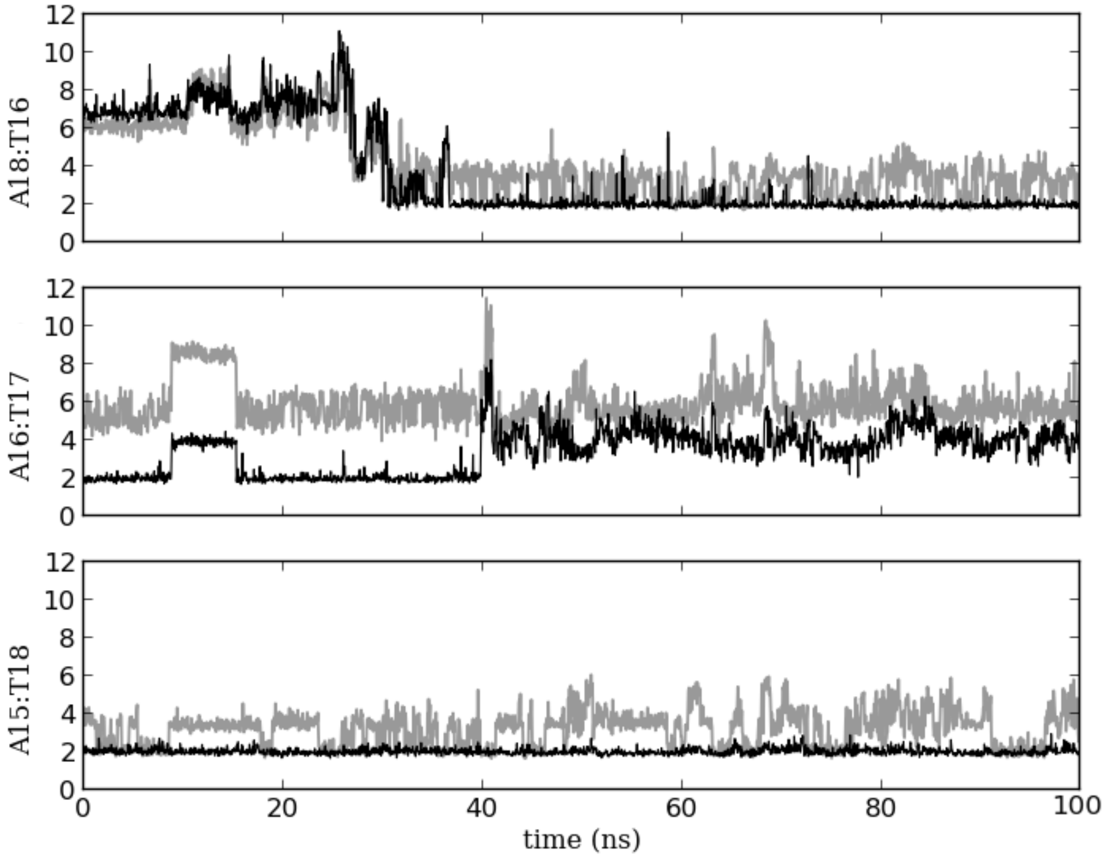

Figure SI-12. Stability of reversed Watson-Crick and Hoogsteen base pairing. Time evolution of two Watson-Crick (A18:T16, A16:T17, top and middle panels)) and one Hoogsteen (A15:T18, bottom panel) base pairings between adenines of the complementary strand and thymines of the outgoing strand during 100 ns molecular dynamics simulation. The initially constructed A16:T17 base pair (middle, N1-H3, black; H61/2H-04, grey, distances in Å) is little stable and completely breaks at 40 ns but new Watson-Crick pairing interactions form at ~37 ns between A18 and T16 (top, same color code), which remain stable during the rest of the simulation. The initially constructed A15:T18 Hoogsteen interaction (bottom, A15:N7–T18:H3, black; A15:H61/2–T18:O2, grey) remains stable during the whole run.

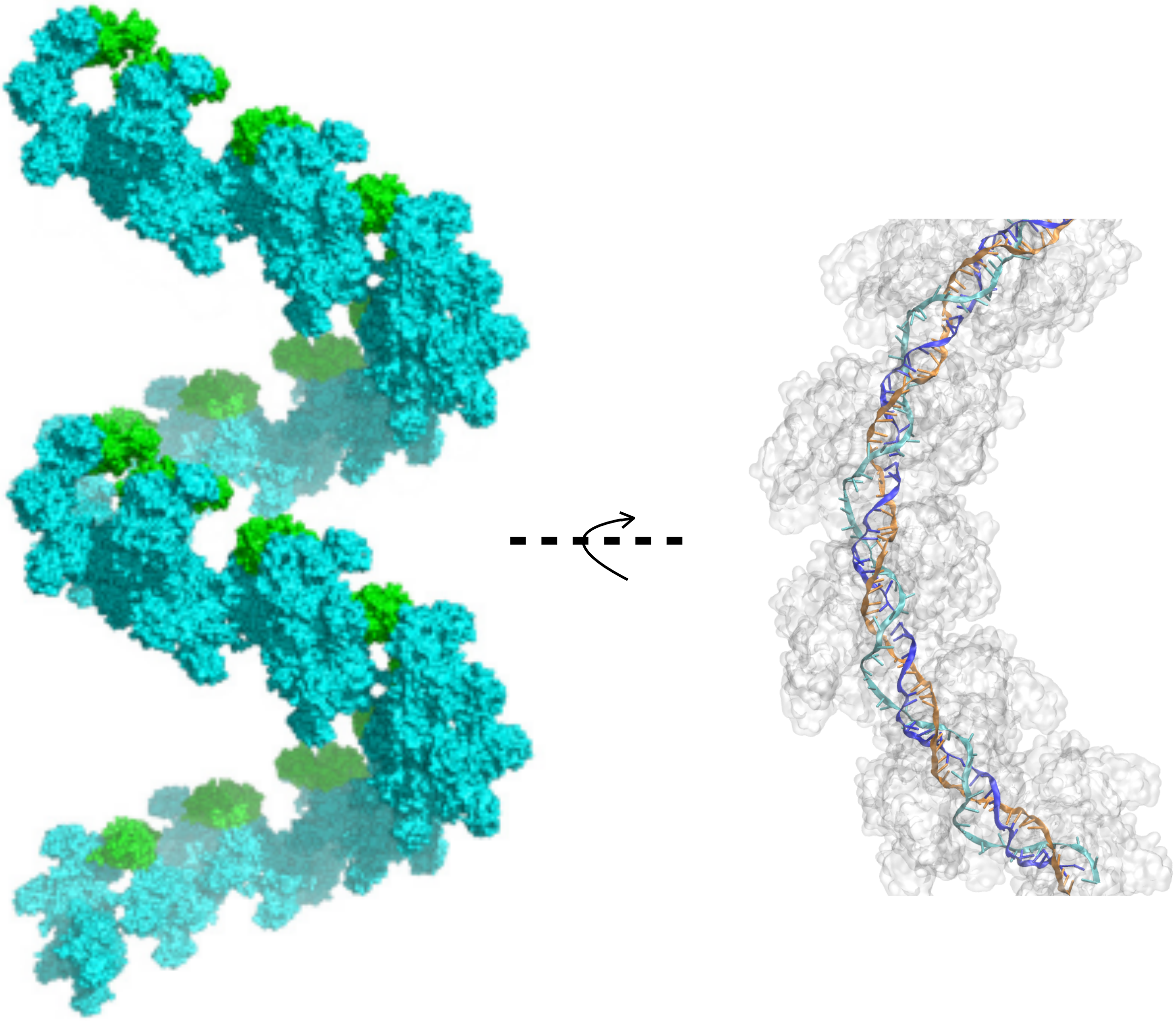

Figure SI-13. Superhelical structure resulting from a periodic distribution of ADP-type interfaces every six monomers in a filament otherwise in the ATP-form, following the Cox model of hydrolysis waves (Cox et al., PloS Biol, 2005, 3, e52). (A) Overall view of the left-handed superhelix; the monomers with an ADP-type upper interface are in cyan. The superhelix contains 59 monomers per super-turn, its pitch is 312 Å and the values of external/internal diameters are respectively 364 and 154 Å. The figure was made with PyMOL (The PyMOL Molecular Graphics System, version 1.3r1 (2010) Schrodinger, LLC, Portland, OR). (B) Accommodation of three DNA strands in one superhelix turn, detailed over 5 helix turns in a top view; the color code for the DNA strands is the same as in Figures SI-1 or SI-8; the protein is represented in transparency.
